# The effectiveness of ultrasound in the detection of fractures in adults with suspected upper or lower limb injury: a systematic review and subgroup meta-analysis

**DOI:** 10.1186/s12873-019-0226-5

**Published:** 2019-01-28

**Authors:** Natalie Champagne, Leila Eadie, Luke Regan, Philip Wilson

**Affiliations:** 1 NHS Grampian, Aberdeen Royal Infirmary, Aberdeen, Scotland, UK; 2 Centre for Rural Health, University of Aberdeen, Inverness, Scotland, UK; 3 NHS Highland, Raigmore Hospital, Inverness, Scotland, UK

**Keywords:** Ultrasound, Ultrasonography, Sonography, Trauma, Fracture, Bone, Diagnostic imaging, Radiology, Adults

## Abstract

**Background:**

The aim of the present review is to assess the effectiveness of ultrasound (US) in the detection of upper and lower limb bone fractures in adults compared to a diagnostic gold standard available in secondary and tertiary care centres (e.g. radiography, CT scan or MRI).

**Methods:**

The review followed PRISMA guidelines and used a database-specific search strategy with Medline, EMBASE and The Cochrane Library plus secondary sources (see supplementary material for completed PRISMA checklist). Diagnostic performance of ultrasound was assessed with a qualitative synthesis and a meta-analysis of two data subgroups.

**Results:**

Twenty-six studies were included (*n* = 2360; fracture prevalence =5.3 % to 75.0%); data were organised into anatomical subgroups, two of which were subjected to meta-analysis. Sensitivity and specificity ranged from 42.11 − 100% and 65.0 − 100%, with the highest diagnostic accuracy in fractures of the foot and ankle. The pooled sensitivity and specificity of US was 0.93 and 0.92 for upper limb fractures (*I*^2^ = 54.7 % ; 66.3%), and 0.83 and 0.93 for lower limb fractures (*I*^2^ = 90.1 % ; 83.5%).

**Conclusion:**

Ultrasonography demonstrates good diagnostic accuracy in the detection of upper and lower limb bone fractures in adults, especially in fractures of the foot and ankle. This is supported by pooled analysis of upper and lower limb fracture subgroups. Further research in larger populations is necessary to validate and strengthen the quality of the available evidence prior to recommending US as a first-line imaging modality for prehospital use.

**Trial registration:**

The protocol is registered with the PROSPERO International register of systematic reviews: ID = CRD42017053640.

**Electronic supplementary material:**

The online version of this article (10.1186/s12873-019-0226-5) contains supplementary material, which is available to authorized users.

## Background

The use of ultrasonography in resource-poor settings has been recommended by the World Health Organisation (WHO) as an achievable healthcare goal, with the caveat that its accuracy relies on the skill of the operator [1]. This is a significant economic limitation to its deployment, as it requires adequately trained personnel to operate the device and interpret the images. Nevertheless, it has already been utilised in prehospital settings with promising results, particularly for the remote triage of traumatic injuries [2].

## Rationale

The focus of trauma ultrasonography has previously been on the validation of the FAST examination in remote settings [3]. This technique initially focussed on the imaging of three abdominal windows and has been extended to include a chest examination (eFAST) [3]. It has been widely validated for the timely assessment and triage of haemodynamically unstable abdominal or thoracic trauma patients [4, 5], and is a core component of Advanced Trauma Life Support algorithms worldwide [5].

However, recent studies have suggested that this application might be expanded to include the identification of bone fractures. Given the potential advantages of ultrasonography in remote and resource-poor settings, the validation of this tool in point-of-care fracture diagnosis could potentially allow timely and appropriate management of fractures in the community.

Although the current evidence for ultrasound-mediated diagnosis of fractures is sparse, a recent meta-analysis conducted by Douma-den Hamer et al. [6] concluded that sonography is reliable in the diagnosis of distal paediatric forearm fractures. Additionally, several studies have suggested that its multi-planar capabilities might make it superior to radiological imaging in the detection of occult or radiographically undetectable stress fractures [7, 8].

The use of sonography in fracture detection has previously been reviewed in paediatric forearm fractures [6], in lower extremity stress fractures [1], in acute extremity fractures [9], and in long bone fractures [10]. However, a systematic review of blinded studies investigating its effectiveness in the identification of upper and lower limb fractures in adults has not yet been conducted and forms the basis for the present review.

## Methods

### Objectives

The aim of the present review is to assess the relative effectiveness of ultrasound in the detection of upper and lower limb fractures in adults compared to the current gold standard for diagnosis (e.g. x-ray, CT scan or MRI).

The PICO model [11] was used to frame the clinical question forming the basis of this review (see Table 1). This framework enables further clarification of the review question by categorising inclusion and exclusion criteria based on the characteristics of **P**atients considered for the study, the **I**ntervention being evaluated, the **C**omparison treatment, and the clinical **O**utcomes being studied [11].

**Table 1:** PICO model

P	Study participants (patients) Diagnostic imaging operators (sonographers, physicians)
I	Point-of-care ultrasound (in Emergency Department, patient bedside, or prehospital setting) For clinically suspected upper or lower limb fracture
C	Gold standard diagnostic imaging (X-ray, CT scan or MRI)
O	Diagnostic accuracy (sensitivity, specificity, negative predictive value, positive predictive value) Patient outcomes (management, comparative time to diagnosis, user perspectives)

Review question following the PICO framework [11]

As the studies included within this review are clinical in nature, the population refers to the study participants. To further assess the use of ultrasound as a diagnostic modality, data relating to the competence and qualifications of the sonographers were extracted where available.

The present review investigates point-of-care ultrasound as a diagnostic modality in a hospital or prehospital setting. All included studies compare sonography to an accepted gold standard diagnostic imaging intervention (radiography, CT scan or MRI). The interpretation of the imaging studies was required to have been blinded for study inclusion.

The primary outcome extracted from the present review is diagnostic accuracy of sonography in the identification of fractures, which is objectively assessed and compared to gold standard imaging using measures of sensitivity, specificity, negative predictive value (NPV) and positive predictive value (PPV).

Secondary outcomes assessed, but not mandatory to study inclusion, relate primarily to the impact of intervention on patients. These included the relative differences in clinical management and time to diagnosis between ultrasonography and the gold standard imaging modality, as well as user perspectives on the use of ultrasonography for fracture diagnosis.

### Eligibility criteria

Screening of eligible studies was conducted based on a predetermined set of inclusion and exclusion criteria (Table 2), which included the parameters of the study question as detailed previously using the PICO framework (Table 1).

**Table 2:** Eligibility criteria

Domain	Inclusion	Exclusion
Study type	Prospective observational study Retrospective observational study Randomised controlled trial Full text Published in a peer reviewed journal	Selected case series Non-clinical study Literature review Conference proceeding Full text not available after request
Participants	Human Adults Clinical setting Blinded ultrasound operators	Non-human subjects Simulated patients Simulated fractures Non-blinded ultrasound operators Non-blinded image interpretation Exclusively paediatric patients Mixed paediatric and adult populations (where paediatric and adult groups are not possible to identify separately)
Setting	Emergency Department Hospital bedside Prehospital setting	No exclusion criteria
Procedure	Diagnostic ultrasonography Diagnostic X-ray OR CT scan OR MRI For clinically suspected upper or lower limb fracture	Therapeutic ultrasonography
Aims/outcomes	Fracture identification Fracture diagnosis Patient management User perspectives	Fracture treatment Bone density assessment

Inclusion and exclusion criteria for study selection in the literature review process

After a preliminary review of the literature related to this topic, the authors decided to focus this review on studies which used ultrasonography to investigate clinically suspected upper or lower limb fractures in adults. As this paper focuses on clinical practice, studies which involved non-human subjects or simulated fractures were excluded. Additionally, studies which included mixed paediatric and adult populations were included where there was separate analysis of diagnostic accuracy for these groups.

To review the diagnostic accuracy of ultrasonography, participant sampling was required to have been consecutive or random. Consequently, papers were screened based on their methodology in order to include only observational studies or randomised controlled trials conducted with appropriate blinding of the diagnostic imaging interpretation.

No limitations were placed on the year or language of the report publication, although a full-text article published in a peer-reviewed journal was required for inclusion. Moreover, older studies were more likely to be excluded based on the method of diagnostic ultrasonography employed. Prior to recent technological advances which have enabled higher resolution imaging using ultrasound [12], it was experimentally used to detect fractures based on its ability to elicit pain at the site of a fracture [13]. As the focus of the present review is on ultrasonography as an imaging modality for diagnostic purposes, studies which used therapeutic ultrasound scans were excluded.

The authors included all studies which reported any of the primary outcomes listed in the PICO framework (see Table 1). Secondary outcome measures were considered separately and did not have an impact on study selection. These included the impact of ultrasonography on patient management, differences in time to diagnosis between ultrasonography and the gold standard modality, and participants’ and investigators’ perspectives relating to ultrasound use in clinically suspected fractures. Studies not reporting objective diagnostic accuracy of ultrasonography in comparison to a reference standard were excluded from the review.

### Database search strategy

One author (NC) conducted a comprehensive search of all relevant articles on 03 January 2017, which was repeated on 18 July 2017, using a database-specific search strategy for each of the following electronic databases: Medline, EMBASE and The Cochrane Library. The search strategy included a combination of multiple iterations of MeSH and keyword terms relating to each component of the research question (see Additional file 1).

The initial literature search yielded 2601 unique potential papers, with a further 53 identified during a search of secondary sources. This search was conducted using the bibliographic references from the included papers obtained in the initial search, discussion with topic experts, and informal searches of Google Scholar and Research Gate databases. Full-text articles were obtained for each source.

### Study selection

Studies acquired through the initial database search with titles judged to be relevant were compiled and managed centrally in Refworks, at which point duplicate studies were removed. The full citations and article abstracts were included in this list and shared between authors to facilitate the screening and final selection of papers for the purposes of the present review.

Initial screening of article titles was conducted during the database search by a single author (NC), and screening of abstracts against inclusion and exclusion criteria was performed by the same person (NC). These processes identified 139 potentially relevant studies, for which full-text articles were obtained and independently screened by two authors (LE, NC).

**Fig. 1:**
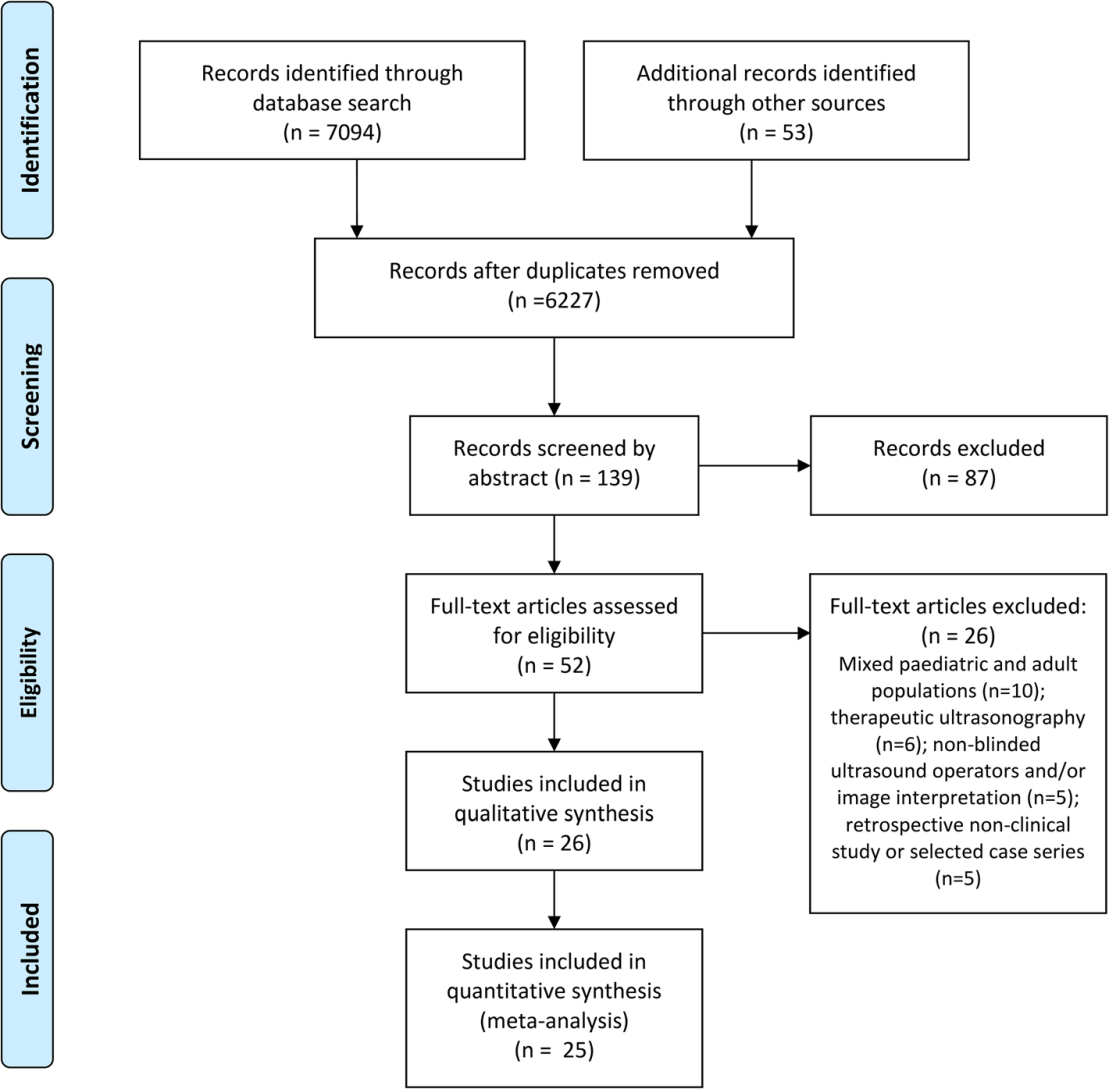
PRISMA flowchart [74]: Outline of search strategy using *MEDLINE*, *EMBASE*, and *The Cochrane Library*, and breakdown of study selection process using inclusion and exclusion criteria

Full-text articles not meeting the inclusion criteria were excluded from the review and the reason for this decision was noted. Exclusion of papers due to mixed paediatric and adult populations, use of therapeutic ultrasonography, non-blinded ultrasound operators, non-blinded image interpretation and non-clinical study methodology generated 26 relevant studies for review (see Fig. 1). Any discrepancy in authors’ decisions regarding study exclusion was resolved by discussion with a third author (PW) and ensured that agreement was reached with respect to the included studies.

### Assessment of bias risk

Following study selection, the 26 trials were appraised to assess their risk of bias using the Critical Appraisal Skills Programme (CASP) Diagnostic Test Study Checklist [14]. This checklist aims to establish the methodological quality of studies by prompting researchers to answer questions relating to its validity (i.e. verification, review and spectrum bias), its results (i.e. the diagnostic accuracy of each intervention arm), and the generalisability of the study results).

**Fig. 2:**
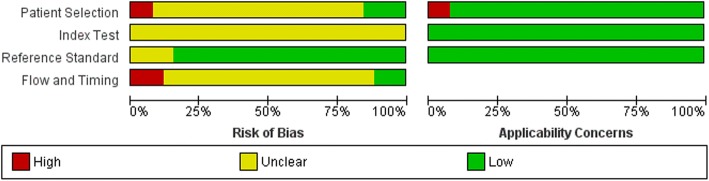
Risk of bias chart. Levels of bias across the 4 domains used by the Cochrane Handbook to establish internal validity [15]. The risk of bias chart summarises the overall bias of all studies, and any applicability concerns (right side of diagram). Figures constructed using *Review Manager*

The critical appraisal of each study was conducted by two independent researchers (LE, NC), and discussion between these authors ensured that agreement is reached where there is any discrepancy of results. The quality of the study protocols was then further analysed following the Cochrane Handbook guidelines for critical appraisal of diagnostic studies [15]. The results from this analysis were listed individually for each study in a ‘risk of bias’ table and summarised to give an overall view of the methodological quality of all included studies in a ‘risk of bias’ graph (see Fig. 2).

### Data collection and extraction

A predefined data extraction form was used by one author (NC) to collect relevant information from the included studies (see Additional file 2). The information fields included on the data collection form were based on the recommendations made by the *Cochrane Handbook for Systematic Reviews of Interventions* [15], and included the following components:

- **Source:** Study ID and review author ID, date review was conducted, citation;
- **Eligibility:** Confirmed based on inclusion/exclusion criteria; reason for study exclusion noted;
- **Methods:** Study design, total study duration, ultrasound operators (profession, experience, blinding), diagnostic image interpreters (profession, experience, blinding), statistical analysis;
- **Participants:** Characteristics of study participants (number, age, sex, setting, country), diagnostic criteria (type of fracture, study inclusion/exclusion criteria);
- **Interventions:** Number of intervention groups, ultrasound group (modality used, intervention details), gold standard imaging (modality used, intervention details);
- **Outcomes:** Outcomes measured (name, definition, unit of measurement);
- **Results:** Diagnostic accuracy (sensitivity, specificity, negative predictive value, positive predictive value), secondary outcomes (patient management, time to diagnosis, user perspectives);
- **Miscellaneous:** Funding source, key conclusions by study authors.

### Data synthesis and analysis

The data were compiled and subjected to both a descriptive and quantitative analysis. As studies used a variety of measures to report diagnostic accuracy, available data was recorded in the ‘calculator’ function of *Review Manager* (RevMan) software (v5.3) to produce similar summary statistics across studies. This allowed pooling of the data and comparison of outcomes across studies using measures of diagnostic test sensitivity and specificity. Meta-DiSc software [16] was used to perform a meta-analysis of the data, and a random effects model was applied due to expected heterogeneity between studies.

Heterogeneity was assessed using the *I*^2^ statistic, which describes the percentage of variability due directly to heterogeneity, with > 50% representing moderate heterogeneity and > 75% indicating high heterogeneity [17]. A sensitivity analysis was performed by removing one study at a time from the pooled analysis to determine whether the results could have been unduly influenced by a single study.

## Results

### Description of studies

A total of 7094 relevant articles were identified in *MEDLINE* (2620 results), *EMBASE* (4259 results), and *The Cochrane Library* (215 results), with an additional 53 studies identified through secondary sources. Exclusion of papers involving non-human studies reduced the yield to 6441 articles, of which 267 were duplicates. The remaining 6227 studies were screened by title to remove studies not meeting the inclusion criteria, producing 139 abstracts for assessment of eligibility. As illustrated in Fig. 1, a total of 52 full-text articles were reviewed as part of this process, 26 of which were included in the present review and qualitative synthesis of results.

### Included studies

The characteristics of the 26 included studies are described in Additional file 3. All trials were prospective observational studies in which participants were subjected to both the intervention and the gold-standard diagnostic modality for the fracture type involved. The control modality was primarily radiography, although CT [12, 18–21] and MRI [22–24] were also employed. In two studies, the authors relied on CT scanning if plain radiography was equivocal [25] or if results were inconsistent between plain x-rays and point-of-care sonography [26].

In total, 2360 participants were analysed across the 26 study populations. These varied considerably in sample size, from 15 to 260 subjects, and consisted of participants recruited consecutively upon presentation to the Emergency Department, with the exception of Dallaudière et al., who conducted their research in a prehospital setting [27], and two papers which recruited participants from specialist units, namely hand surgery [12] and rheumatology [22]. The largest bulk of evidence was produced by research conducted in Turkey [13, 19, 26, 28–33] and other Middle-Eastern countries such as Iran [34, 35] and Israel [24], with the remaining 14 studies spread across continental Europe and the Americas.

Fracture sites varied widely across the included studies, with one paper not reporting outcomes relating to specific fracture types [21]. Fractures of the hand were the most commonly investigated site of injury, with six studies focussed on metacarpal and/or phalangeal fractures [29, 30, 32, 33, 35, 36], and three dealing with fractures of the scaphoid [12, 20, 23]. Other specific injury sites included fractures of the foot and ankle [13, 28, 31, 37, 38], including metatarsal stress fractures [22] and Hill-Sachs injuries [18, 39]. The remaining eight studies focussed on a heterogeneous mixture of upper and lower limb fractures [27, 34] involving the radius and/or ulna [26, 40], humerus and/or femur [25, 41], hip [24] and patella [19].

The primary outcome of interest, diagnostic accuracy of ultrasonography compared to gold standard imaging, was assessed by measures of sensitivity and specificity (reported in all included studies, with the exception of that published by Hedelin et al. [37]) and positive and negative predictive values. Secondary outcomes such as patient outcomes were discussed qualitatively, as the protocols of included studies mandated that patient management be directed by the reference diagnostic standard. However, a number of studies projected the impact of ultrasonography as a first-line imaging modality by estimating the number of x-rays which could have been avoided in the study population [37]. No studies compared the time to diagnosis between ultrasound scans and gold-standard imaging, but one paper noted patient satisfaction scores [13].

### Excluded studies

A total of 26 studies were excluded from the present review on the basis of their full-text articles (listed in Additional file 4). Principal reasons for exclusion were mixed paediatric and adult populations in which the researchers did not separately report outcomes for these groups [7, 42–50], lack of blinding in imaging or interpretation [51–55], and the use of therapeutic instead of diagnostic ultrasonography [56–61]. An additional five papers were excluded based on methodology, with four non-clinical studies [62–65], and one selected case series [66].

### Critical appraisal

The critical appraisal stage of the present review aimed to establish the internal validity of each study using the CASP Diagnostic Test Study Checklist [14], which evaluates each trial protocol on the basis of validity, blinding, interventions and outcome measures. Each of these components can introduce bias, influencing the quality and validity of the study’s results. Two authors (LE, NC) collated the critical appraisals to assess each study’s quality, and these are summarised in a risk of bias chart (Fig. 2) and risk of bias summary constructed using *Review Manager* software and following the Scottish Intercollegiate Guidelines Network (SIGN) guidelines for critical appraisal [67]. The outcomes of the critical appraisals are summarised in Additional file 5.

### Patient selection

In studies evaluating diagnostic test accuracy, selection bias may occur when the method of sampling participants allows researchers to select individual patients for inclusion in the study. This may be prevented by designing a protocol which enrolls all consecutive patients clinically suspected of having the desired condition within a specified time period or a randomised sample of these patients [67].

All included studies enrolled patients consecutively, with most using a convenience sampling design – that is, patients meeting the inclusion criteria were recruited within the specified time period if a researcher or sonographer was present at the time of patient presentation to hospital [19–21, 25, 28, 30–33, 36–38, 41]. Despite randomising patient selection in order to minimize researcher influence, this method of sampling may introduce some degree of selection bias if certain characteristics are overrepresented in patients presenting to hospital between specific working hours. Nine studies did not specify whether recruitment of patients was influenced by staffing [18, 24, 26, 27, 29, 34, 35, 39, 40], and four studies recruited all consecutive eligible patients [12, 13, 22, 23].

The SIGN checklist [67] suggests that case-control methodologies have a tendency to exaggerate diagnostic accuracy, and thus to introduce bias into the study. This may be a source of bias in the papers published by Banal et al. [22] and Lau et al. [40].

With the exception of these potential sources of bias, the study participants were generally deemed to be representative of the target population. The degree of applicability of selected populations was unclear in studies which did not define participant inclusion and/or exclusion criteria [18, 20, 23, 39], and the risk of bias was higher in studies which had more extensive exclusion criteria (which may result in over- or underestimates of diagnostic accuracy). Dallaudière et al. [27] excluded 44 patients due to polytrauma, surgical abdomen, renal colic pain, pulmonary infection, vertebral fracture, phlebitis, orchitis, and cervical swelling. Other studies excluded a variable proportion of eligible patients for reasons such as cognitive impairment [37], pregnancy [38] and incarceration [38].

### Index test

The index test used for fracture diagnosis was a major inclusion criterion of the present review. All studies employed ultrasonography, with point-of-care ultrasonography (PoCUS) being the most common, and only two studies [12, 23] using an alternative (high-spatial-resolution ultrasonography). Potential sources of bias introduced by the index test include lack of blinding of the researcher conducting the test, poorly or undefined diagnostic thresholds, and variability in test execution or interpretation [67]. All included studies had adequate blinding of the index test – that is, the index test was either conducted prior to the reference standard, or by a separate investigator blinded to the results of the reference standard. Consequently, this was not a source of bias in these studies.

With the exception of six studies, the study authors predefined the sonographic features required to make a diagnosis of bone fracture in order to establish some diagnostic threshold in the methodology. However, in the aforementioned six studies [13, 27, 29, 34, 37, 38], there was considerable variability in the detail of reported diagnostic thresholds and some uncertainty with regards to the setting of these thresholds. For five of these studies [13, 27, 29, 34, 38], the methodologies state simply that the presence or absence of fracture was recorded by the sonographer. Dallaudière et al. also state that the number of fragments and distance between these was also recorded [27], and Hedelin et al. allow for results to be recorded as “uncertain/other result” in the case of diagnostic uncertainty [37]. Given that ultrasonography is by its very nature operator-dependent, the lack of predefined diagnostic criteria in the methodology is an important source of bias in these studies.

In addition, the protocols of these studies fail to describe the execution of the index test in a level of detail sufficient to ensure its reproducibility. Although all six papers defined the type of ultrasound machine and the frequency of the probe employed, the planes imaged and the examination protocol was not reported. Any variability in execution of ultrasound examinations, either within or between studies, may introduce bias in interpretation of results and the estimates of diagnostic accuracy [67].

### Reference standard

One of the important sources of bias in the present review is the variability in accuracy of the reference standard for the diagnosis of bone fractures at different sites. The SIGN methodology states that estimates of diagnostic test accuracy in such studies rely on the assumption that the reference standard has a sensitivity of 100% for the condition investigated [67]. Other sources of bias in the methodology relating to the reference standard test include lack of blinding of test execution or interpretation and variability of the diagnostic threshold level across studies [67].

In the present review, the latter items are not a significant source of bias, as blinding occurs across all papers and there are accepted reference standard thresholds for the diagnosis of most bone fractures. However, the accuracy of diagnosis using standard radiography varies greatly depending on the site of the fracture, and therefore is an important consideration when balancing the evidence provided by studies in this review.

Standard radiography was employed as the reference standard for, and has a high sensitivity for diagnosing, fractures of the hand/arm [25, 26, 29, 30, 32, 33, 35, 36, 40], patella [19] and foot/ankle [13, 28, 31, 37, 38], with the exception of metatarsal stress fractures, which are usually radiologically occult at onset [22]. However, Banal et al. avoided introducing bias into their investigation of this fracture type by utilising MRI, which is a highly sensitive modality for the diagnosis of stress fractures [22]. MRI was also used by Safran et al. [24] as the reference standard for hip fractures, for which it has high diagnostic accuracy.

Conversely, radiography has a poorer sensitivity for the detection of Hill-Sachs lesions (between 74 and 93% using the Stryker notch view) [39] and scaphoid fractures, which are often radiologically undetectable at the time of injury [23]. The risk of bias relating to the reference standard used was minimised by the decision of investigators to use more sensitive tests in their methodologies, such as double-contrast CTA [18], non-contrast CT [12, 20] and MRI [22–24]. Herneth et al. also reported radiological findings in their investigation of scaphoid fracture detection, in order to also establish the relative sensitivity of ultrasonography in comparison to this modality [23].

Studies in which the reference standard was a potential source of bias either focussed on fractures at a number of different sites [25, 27, 34] or employed a diagnostic standard with poor sensitivity for the fracture site [39]. In the case of the former, there was some degree of variability in the diagnostic sensitivity of radiography based on the site of each individual patient’s fracture, thus introducing bias into the estimate of diagnostic accuracy of ultrasonography. In the latter, Čičak et al. utilised radiography as the main imaging modality for Hill-Sachs lesion detection, although surgical findings were also reported, and considered the gold-standard [39]. This is a potential source of bias as more accurate diagnostic procedures are available, namely CT-angiography (CTA), which was used by Farin et al. [18].

### Flow and timing

The final domain considered in the present critical appraisal relates to the timing of diagnostic testing and the homogeneity of reference standards and outcomes reporting across the pooled patient population. For the most part, the studies included all patients in the analysis of outcomes and results, with the exception of the paper by Yesilaras et al., which excluded four patients due to loss of sonographer blindness [28]. This may introduce bias in the study if there is some systematic difference between the excluded participants and those included in the final analysis [67].

Although the vast majority of studies employed the same index test and reference standard in all participants, some methodologies allowed for the use of a second reference standard based on the results of index test [18, 25, 39, 68]. For instance, Sivrikaya et al. conducted CT scans on patients whose ultrasound and radiography results were inconsistent [26]. Such variability across the study population may lead to overestimates of sensitivity and specificity of the index test in the final analysis of results.

Finally, bias may be introduced in a study if there is a chance of the participant’s clinical condition changing between the application of the index test and the reference standard. This is most likely if a significant amount of time elapses before both diagnostic tests are conducted. Overall, the timing of the index test and reference standard is very poorly reported, with only 12 of the 26 studies describing an appropriate interval between these tests [12, 20, 22–24, 29, 31–34, 69, 70]. Thus, the risk of bias due to the results being invalidated by test timing is unclear in the remaining 14 papers.

### Effect of interventions

The diagnostic accuracy of point-of-care ultrasonography in the identification of bone fractures was a primary outcome in all studies. Summary tables of primary outcomes extracted from included studies can be found in Additional file 6. Three studies also included secondary outcomes such as patient satisfaction [13], pain [50] and speed of ultrasound examination [36].

### Fractures of the hand

Six studies investigated fractures of the bones in the hand, two of which specifically targeted phalangeal fractures [30, 33], two dealing with metacarpal fractures [29, 32], and the remaining two studies investigating a combination of these fracture sites [35, 71]. Collectively, these studies included 679 patients and ultrasonography identified 177 fractures in the bones of the hand, with fracture prevalence ranging from 26.9 to 46.9%. PoCUS was revealed to have an overall sensitivity ranging from 72.73 to 100%, and specificity between 77.78 and 98.4%.

Sensitivity for the detection of metacarpal fractures [29, 32, 35] with PoCUS ranged from 72.73 to 97.4%, with two of the three studies achieving rates of > 90%. Specificity ranged from 77.78 to 98.28%, with two of three studies achieving rates of > 90%. At this fracture site, PoCUS was found to have a negative predictive value of between 70 to 97.5%, and a positive predictive value of 80 to 97.37%.

The accuracy of phalangeal fracture diagnosis [30, 33, 35] was shown to be comparable, with a sensitivity ranging from 79.3 to 100%, and a specificity of 90 to 98.4%. One of the three studies achieved a sensitivity of > 90% [30], whereas all three studies produced rates of specificity > 90%. Only two studies examining phalangeal fractures reported negative and positive predictive values of ultrasonography for fracture detection [8, 33], with rates ranging between 92.68 to 93.1% and 71.8 to 78.95% respectively.

Finally, the study by Tayal et al. [36] pooled results from metacarpal and phalangeal fractures, which were detected with a sensitivity of 90% and a specificity of 98%. The investigators also recorded data relating to the speed of ultrasound examination, which was rated as being rapid (< 5 min) by 73% of physicians and average (> 5 min) by the remaining 27% of clinicians.

### Fractures of the scaphoid

Three papers examined the use of ultrasonography for the detection of scaphoid fractures in a pooled population of 101 patients with acute wrist trauma. Interestingly, two of the studies excluded patients with positive radiographic findings, aiming to focus only on the detection of occult fractures [12, 66], whilst the third study included any clinically suspected scaphoid fracture [23]. Additionally, the type of ultrasonography employed varied across the studies, with two papers employing high-spatial-resolution ultrasound [12, 23] and one using point-of-care ultrasonography [10].

Gold standard imaging (either CT or MRI) identified a fractured scaphoid in 27 participants, and the prevalence of fractures across patient populations ranged from 21 to 60%. Sensitivity of ultrasonography for the detection of these fractures ranged from 78 to 100%, with the two studies focussing on occult scaphoid fractures achieving rates > 90%. In contrast, specificity was lower overall, with rates between 71 and 100%, and only one study reported rates > 90% [23].

All three studies also reported negative and positive predictive values for this diagnostic test, which ranged from 75 to 100% and 46 to 100% respectively. The study by Fusetti et al. recorded sonographic findings as a range of clinical suspicion (high, intermediate, and low), based on the number of imaging criteria identified on ultrasound [12]. In contrast, the sonographic findings reported in the other two studies consisted of the presence or absence of scaphoid fracture on imaging. Interestingly, the former over-reported scaphoid fractures, with seven false-positive findings (high index of suspicion), whereas there were no false-positives reported in the latter two papers.

### Fractures of the foot and ankle

Six of the included papers focussed on bones in the foot and ankle, four of which investigated fractures of any bone in the foot and/or ankle [13, 31, 36, 37]. The remaining two studies focussed specifically on 5th metatarsal fractures [70] and metatarsal stress fractures [22]. Altogether, these studies included 670 participants and identified a total of 189 fractures, with fracture prevalence ranging from 15.3 to 42%.

In fractures of the foot and/or ankle, PoCUS had a reported sensitivity ranging from 87.3 to 100%, and a specificity between 85.9 and 100%, with three of the five studies achieving sensitivities > 90% and two of the five reporting specificities above this threshold. Negative and positive predictive values for ultrasonography were reported in two of the four studies and were uniformly > 90% (NPV was 100% for both studies, and PPV ranged from 95.2 to 100%). Tollefson et al. also reported that ultrasonography was well-tolerated by all participants [38], while Ekinci et al. reported that 95% of all patients recruited within their study would prefer new trauma to be diagnosed by means of ultrasonography in the future, and that 3.3% of patients had no stated preference [13].

The study by Yesilaras et al. described a sensitivity of 97.1% and a specificity of 100% for ultrasound diagnosis of 5th metatarsal fractures, with all 33 fractures detected by PoCUS and a single false-negative finding reported [28]. In contrast, Banal et al. investigated the use of ultrasonography in the detection of metatarsal stress fractures and found a sensitivity and specificity of 83.3 and 75.9% respectively [22]. Eleven of thirteen fractures were detected, with 2 false-negative and 7 false-positive findings, a negative predictive value of 91.7% and a positive predictive value of 58.8%.

### Fractures of the upper limb

Seven studies investigated the use of ultrasound in the diagnosis of upper limb bone fractures. There were 689 participants with 295 fractures across these studies, and the fracture prevalence ranged from 13.7 to 70.2%. Three of the studies classified the fracture types according to specific bones affected, namely the distal radius, humerus, and radius/ulna [40, 68, 69], while the remainder referred to general anatomical sites of injury (i.e. “upper extremity”, “wrist”).

The overall sensitivity of ultrasonography in the detection of upper limb fractures ranged from 42.11 to 100%, with five of the seven studies achieving rates > 90%. The reported specificity was higher, ranging from 83 to 100%, again with five studies achieving rates > 90%. Javadzadeh et al. recorded the lowest sensitivity, at 42.11% for the detection of wrist fractures [35], and Bolandparvaz et al. had similarly poor sensitivity in the detection of upper limb fractures, at a reported 55.5% [34].

Three of the papers also recorded negative and positive predictive values for ultrasonography, which ranged from 73 to 93.3% and 57.14 to 93.44% respectively. Interestingly, both the highest and the lowest PPVs were reported within the same study at two different fracture sites (lowest in the detection of wrist fractures and highest in the detection of distal forearm fractures) [35].

Three studies also calculated inter- or intra-rater reliability scores, which refer to the degree of agreement among all sonographers, or between repeated applications of ultrasound by a single tester, respectively. In the study by Bolandparvaz et al., interrater reliability was assessed as being average at three fracture sites (upper limb long bones, upper limb joints and lower limb long bones), and relatively weak in the detection of lower limb joint fractures [34]. Lau et al. reported much higher levels of interrater reliability (*κ* = 0.86), and high scores for intra-observer reliability (*κ* = 0.96 for the orthopaedist and *κ* = 0.85 for radiologists) [40].

### Fractures of the lower limb

The six studies focussing on fractures of the lower limb involved a total of 438 patients, and investigated various sites of fracture including the femur [41, 69], patella [19] and hip [24]. The fracture prevalence ranged from 5.3 to 75.0%, and the overall sensitivity and specificity of ultrasonography in fracture diagnosis varied between 75 to 100% and 65 to 100% respectively. Four of the six studies achieved a sensitivity > 90%, and two of the six reported specificities > 90%. Negative and positive predictive values were reported in four of the studies, and rates ranged from 80 to 100% and 59 to 93.8% respectively.

Safran et al. investigated the use of ultrasonography in the detection of occult hip fractures, and found that PoCUS had a sensitivity of 100%, but a poor specificity (65%) [24]. In contrast, Kilic et al. reported that ultrasonography had a good diagnostic accuracy in the diagnosis of patellar fractures, with a sensitivity of 93.3% and a specificity of 94.8% [19]. The two studies investigating the use of ultrasonography in the diagnosis of femoral fractures found comparable results, with sensitivities and specificities > 80% [41, 69].

### Hill-Sachs lesions

Two studies investigated the use of ultrasonography to identify Hill-Sachs lesions [18, 39], which is an eponymous name for posterolateral humeral head compression fractures, typically due to recurring anterior shoulder dislocations. The current gold standard for diagnosis is the Stryker notch view, which is a radiographic projection that has an estimated diagnostic accuracy of 74–93% [39]. These two studies investigated a suspected 147 Hill-Sachs lesions, using the Stryker notch view as the reference standard for establishing diagnostic accuracy. Both studies reported good diagnostic accuracy of ultrasonography, with sensitivities ranging from 91 to 96%, and specificities of 95 to 100%.

### Quantitative analysis of primary outcomes

The data were pooled for quantitative analysis into subgroups according to the fracture site. One study was excluded from the meta-analysis as data were insufficient for the combined analysis [21]. Four studies stratified their data according to the site of injury, and the appropriate subgroups were thus included in both combined analyses [25, 27, 34, 41]. The meta-analysis showed that ultrasonography has an overall high sensitivity and specificity in the identification of fractures, with a pooled diagnostic odds ratio of 139.22 and 98.53 for upper and lower limb fractures respectively (Additional files 7 and 8).

**Fig. 3:**
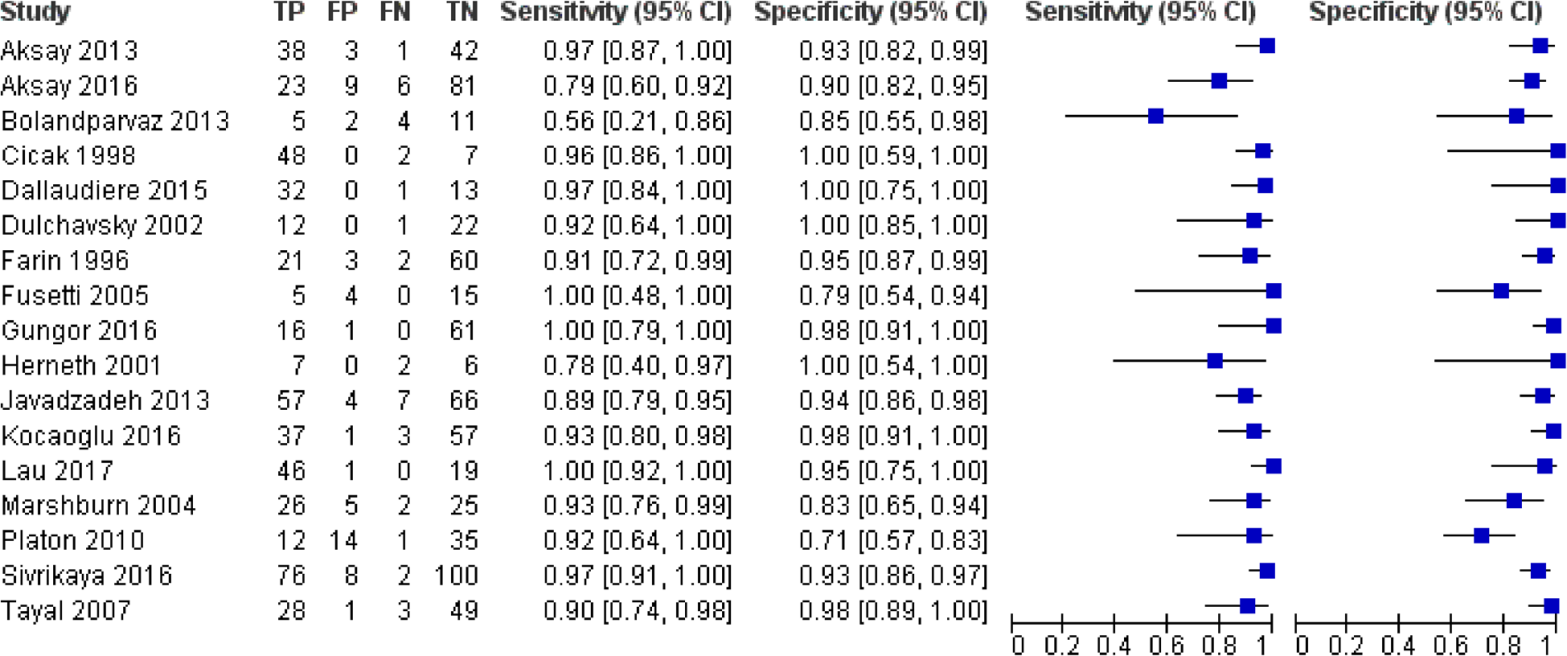
Forest plot for upper limb fractures: Pooled diagnostic accuracy summary across studies assessing the sensitivity and specificity of ultrasonography for fractures of the upper limb. Figure constructed using *RevMan*

In the pooled data for the upper limb fractures (Fig. 3), the sensitivity of ultrasonography for fracture detection was > 75% in all but one study [34], and all studies except that conducted by Platon et al. reported specificities > 75% [20]. Pooled values for sensitivity and specificity were 0.93 and 0.92 respectively, with *I*^2^ statistics of 54.7 and 66.3%. This corresponds to moderate heterogeneity between studies for both measures. The pooled diagnostic odds ratio (DOR) of 145.46 suggests high diagnostic test accuracy, as it consists of a combined summary estimate of sensitivity and specificity, and the *I*^2^ statistic associated with this measure indicates low heterogeneity (I^2^ = 46.4%).

**Fig. 4:**
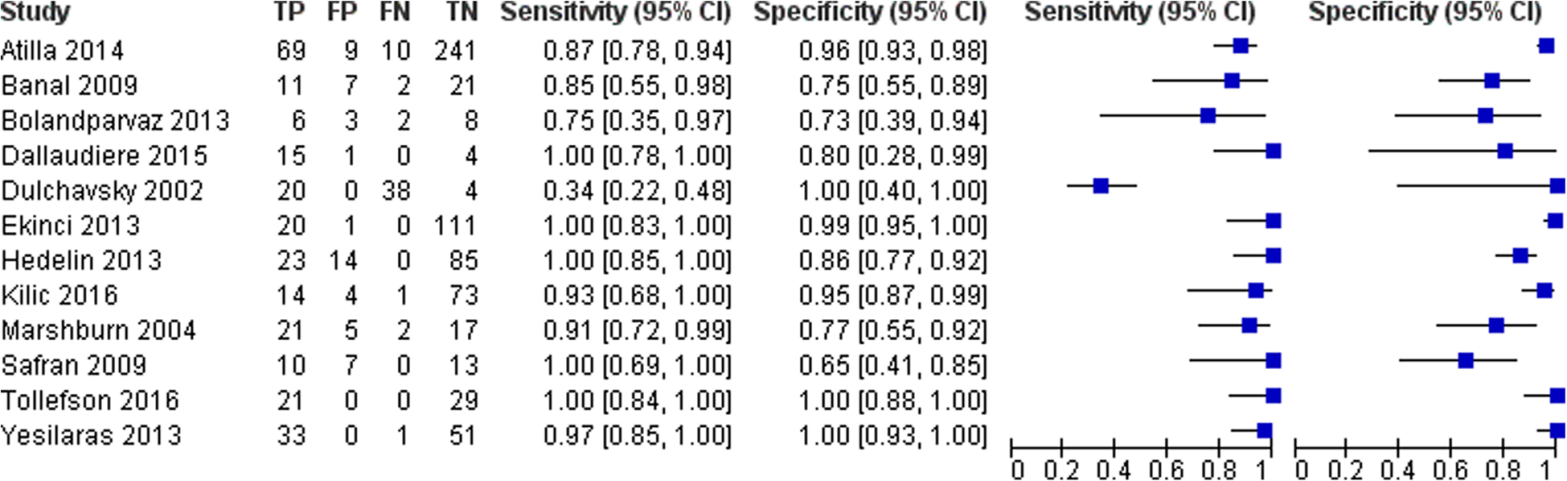
Forest plot for lower limb fractures: Pooled diagnostic accuracy summary across studies assessing the sensitivity and specificity of ultrasonography for fractures of the lower limb. Figure constructed using *RevMan*

The pooled data for the lower limb fractures (Fig. 4) show similar trends, although there is a slight decrease in overall diagnostic accuracy for ultrasonography. The study by Dulchavsky et al. reports a sensitivity of 0.34, which contributes to an overall pooled sensitivity of 0.82, with an *I*^2^ statistic of 90.1% indicating high heterogeneity of pooled studies [41]. In terms of specificity, only Bolandparvaz et al. report a value below 75% [34], and the pooled specificity is 0.93 with an *I*^2^ statistic of 83.5%. The diagnostic odds ratio of 98.53 is high, although considerably lower than that seen in the pooled data from the upper limb fractures, with a moderate heterogeneity of *I*^2^ = 61.9%.

To evaluate the effect of individual studies, a one-by-one sensitivity analysis was conducted. There was no marked change in the pooled sensitivity, specificity, DOR or 95% confidence interval (CI) for the upper limb subgroup of studies, indicating that no individual study influenced the pooled results. For the lower limbs subgroup, there was a marked change both in the pooled sensitivity and the 95% CI for sensitivity measures with the removal of the data from Dulchavsky et al. [41], which increased to 0.93 (0.89–0.96) with an *I*^2^ statistic of 52.1%, indicating substantially reduced heterogeneity with the removal of this study from the analysis.

### Secondary outcome measures

No studies reported outcomes relating to the impact of ultrasonography on patient management or recorded the time to diagnosis with ultrasound or the gold standard diagnostic modality. For ethical approval to have been given prior to study commencement, investigators stated that patient management was required to be guided by the results of the reference standard imaging and the examination findings, as per the usual protocol in each individual secondary care centre.

Users’ perspectives relating to ultrasound for fracture detection was recorded in some form in three studies [13, 26]. Ekinci et al. reported that 95% of patients would prefer ultrasonography to be used in a case of new trauma, whereas 1.7% would not, and 3.3% had no stated preference [13]. No patients reported pain or discomfort during ultrasonography in the study conducted by Weinberg et al. [21]. Finally, physicians’ perspectives on the use of ultrasonography for fracture detection were recorded by Tayal et al., who asked clinicians to rate the speed of examination as rapid (< 5 min), average (5–10 min) or prolonged (> 10 min). This study reported that 73% of physicians rated the speed of examination as rapid, and the remaining 27% stated that it was average [36].

## Discussion

Point-of-care ultrasonography has the potential to become a replacement or a triage test in the identification of bone fractures, depending on the specific site of injury. Although plain radiography has a high sensitivity in the diagnosis of upper and lower limb fractures, there are important challenges associated with accessing this technology in remote environments. Consequently, ultrasonography is increasingly being considered as a first-line modality in the primary response to emergency situations. Furthermore, while radiography is the most commonly used diagnostic tool for the imaging of bony injuries, it has relatively poor diagnostic accuracy for certain fracture types (i.e. scaphoid). Accessibility challenges are exponentially increased when it comes to more accurate imaging techniques such as CT and MRI, and ultrasound may present a viable alternative to radiography in these patients.

The present literature review included 26 studies recruiting a total of 2360 patients, of which 18 reported sensitivity rates > 90% and 16 specificity rates > 90%. Pooled results of the meta-analyses demonstrated that ultrasonography had good diagnostic accuracy for fracture detection in adults, with a sensitivity of 93% and specificity of 92% for the upper limb, and a sensitivity of 82% and specificity of 93% for the lower limb. However, the use of ultrasonography for fracture detection in adults is a relatively new field of study and, although the evidence collected within this review is promising, further research in larger populations is required to support its use in clinical practice.

### Summary of evidence

The present review was tasked with evaluating the quality and the strength of the evidence supporting the use of ultrasonography in the diagnosis of upper and lower limb fractures in adults, in order to assess its potential applications in rural healthcare settings. For ultrasonography to be considered a viable initial alternative in practice, there must be evidence of equivalent or superior diagnostic accuracy compared to a current accepted reference standard. Overall, this systematic review presents evidence that point-of-care ultrasonography has a high diagnostic accuracy in this application.

In the subgroup analysis of results, the authors identified the foot and ankle as the site of highest sensitivity and specificity across multiple studies, with values ranging from 85.9 to 100% and 86.4 to 100% respectively. Additionally, the data from all five included studies noted positive and negative predictive values superior to 90%. This is consistent with previous reviews of the literature pooling data from paediatric and adult populations, which reported ultrasonography as being most accurate in detecting fractures of the ankle [10]. However, the authors note that one the papers reporting on fractures at this anatomical site was assessed as having a high risk of selection bias, due to strict exclusion criteria applied to a consecutive convenience sampling method [38]. Single-study data also suggested that sonography had a good diagnostic accuracy in the detection of patellar fractures [19].

Ultrasonography also presents a reasonable alternative to radiography in the diagnosis of scaphoid and metatarsal stress fractures, and Hill-Sachs lesions. Fractures at these anatomical sites are often radiographically occult at the time of injury, and a more accurate diagnostic test might facilitate the initial screening of patients. While other imaging modalities such as CT and MRI have been shown to accurately diagnose these injuries, these have significant limitations. Therefore, as ultrasonography shows relatively high sensitivity for these types of fracture across the included trials, it might be a safe method of screening out uninjured patients prior to employing more expensive and/or invasive tests.

### Limitations

Trials which used ultrasonography to image multiple anatomical sites or which recruited smaller participant populations reported lower diagnostic accuracies. The former is a finding that has been previously reported in the literature [10], and suggests that focussed sonographer training on specific anatomical regions may result in greater accuracy due to the learning curve associated with imaging different bones. One of the limitations of the present review is the fact that several of the included studies had small sample sizes. The influence of sample size on diagnostic accuracy highlights the imprecision of small studies and supports the authors’ recommendations that future research be conducted in larger groups with a more focussed anatomical region of interest.

Other limitations of the included studies relate to methodologic concerns across multiple studies. There was an increased risk of selection bias in all studies as a result of the convenience sampling methodology employed by a few motivated and trained clinicians. Additionally, ultrasonography is a user-dependent imaging modality, and few studies measured the effect of sonographer experience and training on the reliability of their imaging. Intra- and interrater reliability were infrequently reported, with only three of the 26 included studies noting values for these [28, 34, 40].

### Future research

The use of ultrasonography for fracture detection has most recently been reviewed in 2017 by Chartier et al. [10], and has previously been reviewed in 2013 [9] and 2016 [6]. In addition to seven articles that had not been published at the time of the most recent review [19, 26, 29, 30, 33, 38, 40], the authors found 11 other papers [12, 18, 20, 22–24, 28, 32, 35, 36, 39] that were not included in the review. To the authors’ knowledge, this is the first review of the literature pertaining to the diagnostic use of ultrasonography in solely adult populations. The present review reaches similar conclusions, that ultrasonography is a useful adjunct in the identification of upper and lower limb fractures and supports previous authors’ conclusions that further research is required to strengthen the evidence of its efficacy in this role.

Musculoskeletal ultrasonography has been widely validated as a diagnostic modality for fractures in paediatric populations [72], and future studies should focus on expanding the body of evidence in other age groups, particularly in adults and in the elderly. The use of PoCUS in paediatric patients has been particularly appealing due to its potential to limit unnecessary radiography, and thus reduce exposure to ionising radiation. This suggests a potential application of this diagnostic modality in other susceptible populations, for instance in pregnancy. Future research in larger patient populations focussed on the identification of fractures at specific anatomical sites is warranted.

As it is a portable and relatively inexpensive tool, it also has potential applications in rural and emergency settings. A recent study by Blaivas et al. found that PoCUS “dramatically altered” the management of trauma patients in remote settings, enabling several patients to avoid a risky evacuation to a secondary or tertiary care setting [73]. Future studies should consider the level of training and experience of sonographers in interpreting musculoskeletal ultrasounds, and the effect of variability in training on the reliability of reported results. The development of standardised training to enable healthcare professionals to become proficient in this skill would facilitate its use as a screening tool to identify patients who require additional imaging or radiography and/or transfer to more definitive medical care. Developing an algorithm which would allow first responders to perform initial diagnostic imaging relies on the validation of PoCUS as a highly sensitive test for bone fractures, as it requires confident exclusion of healthy individuals (with a low proportion of false-negatives).

## Conclusion

The current evidence is too limited to support the use of ultrasonography as an initial diagnostic tool for fractures of the upper and lower limb in adults. However, the 26 included prospective studies consistently report good diagnostic accuracy characteristics for the diagnosis of bone fractures in the studied population, and future research may enable its widespread application in this practice. It has the potential to become the primary imaging modality used in remote and rural settings to establish the need for definitive medical transfer and may replace radiography in the initial screening of scaphoid fractures, metatarsal stress fractures, and Hill-Sachs lesions. Future studies, ideally conducted as RCTs, are required to establish training and education standards, and to assess the feasibility and safety of PoCUS as an alternative to radiography.

## Additional files

Additional file 1: Search Strategy. Detailed database-specific search strategy. (PDF 3015 kb)

Additional file 2: Data extraction form. Predefined data extraction form used to collect data from included studies. (PDF 1417 kb)

Additional file 3: Characteristics of Included Studies. Summary of included studies’ features including: location, number of participants, injury site/modalities studied and outcomes measured. (PDF 2085 kb)

Additional file 4 : Characteristics of Excluded Studies. List of studies excluded at the stage of full-text article review, indicating the reason for study exclusion from the systematic review. (PDF 2357 kb)

Additional file 5: Summary of Critical Appraisals. Individual summary of critical appraisal of included studies following the SIGN methodology checklist. (PDF 3009 kb)

Additional file 6: Summary of Study Results. Summary tables of primary outcomes extracted from included studies (PDF 3170 kb)

Additional file 7: Meta-analysis of pooled upper limb fracture data. Meta-analysis tables produced from the pooled subgroup data relating to upper limb fracture detection. (PDF 268 kb)

Additional file 8: Meta-analysis of pooled lower limb fracture data. Meta-analysis tables produced from the pooled subgroup data relating to lower limb fracture detection. (PDF 269 kb)

## Abbreviations

CASP: Critical Appraisals Skills Programme
CI: Confidence interval
CTA: CT angiography
DOR: Diagnostic odds ratio
FAST: Focused Assessment with Sonography for Trauma
NPV: Negative predictive value
PoCUS: Point-of-care ultrasonography
PPV: Positive predictive value
RevMan: Review Manager
SIGN: Scottish Intercollegiate Guidelines Network
US: Ultrasound
WHO: World Health Organisation
