# Supplementary material for: The effectiveness of ultrasound in the detection of fractures in adults with suspected upper or lower limb injury: a systematic review and subgroup meta-analysis
Source: BMC Emerg Med. 2019 Jan 28;19:17. doi: 10.1186/s12873-019-0226-5 (PMC6350304; doi:10.1186/s12873-019-0226-5)
Supplement: Supplementary file 1 — Search Strategy. Detailed database-specific search strategy. (PDF 3015 kb) [file 12873_2019_226_MOESM1_ESM.pdf]

Additional file 1: Search Strategy:

Search strategy: [Concept 1] AND [(Concept 2) OR (Concept 3)] AND [Concept 4]

DATABASE	CONCEPT 1<br>ULTRASOUND	CONCEPT 2<br>X-RAY	CONCEPT 3<br>CT SCAN	CONCEPT 4<br>FRACTURE
**MEDLINE**	1. sonograph*.mp<br>2. ultrasonography/<br>3. ultrasonography<br>4. exp Ultrasonography/<br>5. (ra and us).fs<br>6. ultraso*.mp	1. exp X-Rays/<br>2. exp Radiography/<br>3. (ra and us).fs<br>4. x-rays/<br>5. x-ray*.mp<br>6. radiography/<br>7. radiograph*.mp	1. computed tomography.mp<br>2. ct.mp<br>3. ct scan.mp<br>4. Tomography, X-ray Computed/<br>5. exp Tomography, X-ray Computed/	1. fractures, Bone/<br>2. fractur*.mp<br>3. exp Fractures, Bone/
**EMBASE**	1. ultraso*.mp<br>2. portable ultrasound scanner/<br>3. ultrasound/<br>4. ultrasound scanner/<br>5. sonograph*.mp<br>6. ultrasonography.mp<br>7. exp ultrasound/	1. exp x-ray/<br>2. exp radiography/<br>3. x-rays/<br>4. x-ray*.mp<br>5. radiography/<br>6. radiograph*.mp	1. computed tomography.mp<br>2. ct.mp<br>3. ct scan.mp<br>4. computer assisted tomography/<br>5. exp computer assisted tomography/	1. exp fracture/<br>2. fractur*.mp<br>3. fracture/
**COCHRANE LIBRARY**	1. [mh Ultrasonography]<br>2. [mh Ultrasound, Diagnostic]<br>3. [mh Ultrasounds, Diagnostic]<br>4. [mh Sonography, Medical]<br>5. ultrason*<br>6. sonograph*	1. [mh X Ray Film]<br>2. [mh X Rays]<br>3. [mh X-Ray Film]<br>4. [mh Radiography]<br>5. X-ray*<br>6. X*ray<br>7. radiograph*	1. [mh Tomography Scanners, X-Ray Computed]<br>2. CT scan*<br>3. Computed tomograph*	1. [mh Fractures, Bone]<br>2. [mh Fractures, Closed]<br>3. fractur*
