# Supplementary material for: The effectiveness of ultrasound in the detection of fractures in adults with suspected upper or lower limb injury: a systematic review and subgroup meta-analysis
Source: BMC Emerg Med. 2019 Jan 28;19:17. doi: 10.1186/s12873-019-0226-5 (PMC6350304; doi:10.1186/s12873-019-0226-5)
Supplement: Supplementary file 2 — Data extraction form. Predefined data extraction form used to collect data from included studies. (PDF 1417 kb) [file 12873_2019_226_MOESM2_ESM.pdf]

Additional file 2: Data extraction form:

a) Source:

Full study citation
Study ID *(e.g. Smith 2001)*
Review author ID (date form completed)
Link to full text

b) Study eligibility:

Study characteristics	Inclusion criteria	Yes	No	Unclear	Location in text
Type of study	Prospective observational study<br>Full text				
Participants/setting	Human<br>Patients present (clinical study)				
Outcome measures	Diagnostic accuracy				
Decision	☐ Included<br>☐ Excluded				
Reason for exclusion	*Study type*<br>☐ Selected case series<br>☐ Non-clinical study<br>☐ Literature review<br>☐ Conference proceeding<br>☐ Full text not available<br>*Procedure*<br>☐ Therapeutic ultrasonography<br>*Aims/outcomes*<br>☐ Fracture treatment<br>☐ Bone density assessment	*Participants*<br>☐ Non-human subjects<br>☐ Simulated patients<br>☐ Simulated fractures<br>☐ Non-blinded ultrasound operators<br>☐ Non-blinded image interpretation<br>☐ Exclusively paediatric patients<br>☐ Mixed paediatric and adult populations (where adult and paediatric groups cannot be separately identified)				
Notes					

DO NOT PROCEED IF STUDY IS EXCLUDED FROM REVIEW

3. Data extraction:

<table>
<tr><td colspan="3">c) Methods:</td></tr>
<tr><td colspan="2">Criteria</td><td>Description</td></tr>
<tr><td colspan="2">Study design</td><td></td></tr>
<tr><td colspan="2">Total study duration</td><td></td></tr>
<tr><td colspan="2">Ultrasound operators</td><td></td></tr>
<tr><td colspan="2">Diagnostic image interpreters</td><td></td></tr>
<tr><td colspan="2">Statistical data analysis</td><td></td></tr>
<tr><td colspan="3">d) Participants:</td></tr>
<tr><td colspan="2">Criteria</td><td>Description</td></tr>
<tr><td rowspan="5">Participant characteristics</td><td>Total number</td><td></td></tr>
<tr><td>Age</td><td></td></tr>
<tr><td>Sex</td><td></td></tr>
<tr><td>Setting</td><td></td></tr>
<tr><td>Country</td><td></td></tr>
<tr><td rowspan="3">Diagnostic criteria</td><td>Type of fracture</td><td></td></tr>
<tr><td>Inclusion criteria</td><td></td></tr>
<tr><td>Exclusion criteria</td><td></td></tr>
<tr><td colspan="3">e) Outcomes:</td></tr>
<tr><td colspan="2">Outcomes measured (name, definition, unit of measurement)</td><td></td></tr>
<tr><td colspan="3">f) Results:</td></tr>
<tr><td colspan="2">Primary outcomes</td><td>Description</td></tr>
<tr><td colspan="2">Sensitivity</td><td></td></tr>
<tr><td colspan="2">Specificity</td><td></td></tr>
<tr><td colspan="2">Negative predictive value</td><td></td></tr>
<tr><td colspan="2">Positive predictive value</td><td></td></tr>
<tr><td colspan="2">Secondary outcomes</td><td></td></tr>
<tr><td colspan="2">Patient management</td><td></td></tr>
<tr><td colspan="2">Comparative time to diagnosis</td><td></td></tr>
<tr><td colspan="2">User perspectives</td><td></td></tr>
<tr><td colspan="3">f) Miscellaneous</td></tr>
<tr><td colspan="2">Criteria</td><td>Description</td></tr>
<tr><td colspan="2">Funding source</td><td></td></tr>
<tr><td colspan="2">Key conclusions by study authors</td><td></td></tr>
</table>
