# Supplementary material for: The effectiveness of ultrasound in the detection of fractures in adults with suspected upper or lower limb injury: a systematic review and subgroup meta-analysis
Source: BMC Emerg Med. 2019 Jan 28;19:17. doi: 10.1186/s12873-019-0226-5 (PMC6350304; doi:10.1186/s12873-019-0226-5)
Supplement: Supplementary file 3 — Characteristics of Included Studies. Summary of included studies’ features including: location, number of participants, injury site/modalities studied and outcomes measured. (PDF 2085 kb) [file 12873_2019_226_MOESM3_ESM.pdf]

Additional file 3: Characteristics of Included Studies:

Study (ref)	Location	(n)	Injury site	Intervention	Control	Outcomes
Aksay (40)	Turkey	119	Proximal and middle phalanx	PoCUS	Radiography	Sensitivity; specificity; PPV; NPV; LR(+); LR(-)
Tollefson (45)	USA	50	Foot and/or ankle	PoCUS	Radiography	Sensitivity; specificity; NPV; PPV
Aksay (39)	Turkey	81	5th metacarpus	PoCUS	Radiography	Sensitivity; specificity; PPV; NPV; LR(+); LR(-)
Atilla (38)	Turkey	246	Ankle and/or foot	PoCUS	Radiography	Sensitivity; specificity
Yesilaras (35)	Turkey	84	5th metatarsus	PoCUS	Radiography	Sensitivity; specificity; LR(+); LR(-); intraclass correlation
Bolandparvaz (41)	Iran	80	Long bone	PoCUS	Radiography	Sensitivity; specificity; PPV; NPV; interrater reliability
Ekinci (19)	Turkey	131	Foot and/or ankle	PoCUS	Radiography	Sensitivity; specificity; PPV; NPV; patient satisfaction
Platon (27)	Switzerland	62	Scaphoid	PoCUS	CT scan	Sensitivity; specificity; PPV; NPV; high vs. low-risk fractures
Weinberg (28)	USA	212	Not specified	PoCUS	Radiography[1]	Sensitivity; specificity; LR(-); LR(+);pain; reliability
Safran (31)	Israel	30	Hip	PoCUS	MRI	Sensitivity; specificity; NPV; PPV
Tayal (43)	USA	78	Metacarpus; phalanx	PoCUS	Radiography	Sensitivity; specificity; PPV; NPV; speed of examination
Fusetti (18)	Switzerland	24	Scaphoid	HSR-US	CT scan	Sensitivity; specificity; PPV; NPV; index of suspicion
Marshburn (32)	USA	58	Humerus; femur	PoCUS	Radiography[2]	False-positive; false-negative; sensitivity; specificity
Herneth (30)	Austria	15	Scaphoid	HSR-US	MRI	Accuracy; sensitivity; specificity; PPV; NPV
Čičak (46)	Croatia	61	Shoulder (Hill-Sachs lesion)	PoCUS	Surgical findings	Sensitivity; specificity; accuracy; false-negative

[1]CT scan used as diagnostic modality comparison for skull and mandible fractures (gold standard)
[2]CT scan used if plain radiography was equivocal

Study (ref)	Location	(n)	Injury site	Intervention	Control	Outcomes
Farin (25)	Finland	86	Shoulder (Hill-Sachs lesion)	PoCUS	Double-contrast CTA	Sensitivity; specificity; accuracy
Banal (29)	France	37	Metatarsus (stress #)	PoCUS	MRI	Sensitivity; specificity; PPV; NPV; LR(+); LR(-)
Gungor (37)	Turkey	45	Distal phalanx	PoCUS	Radiography	False-positive; false-negative; sensitivity; specificity
Kocaoğlu (36)	Turkey	96	Metacarpus	PoCUS	Radiography	Sensitivity; specificity; PPV; NPV
Sivrikaya (33)	Turkey	90	Radius; ulna	PoCUS	Radiography[3]	Sensitivity; specificity; LR(+); LR(-)
Kilic (26)	Turkey	92	Patella	PoCUS	CT scan	Sensitivity; specificity
Javadzadeh (42)	Iran	260	Distal forearm; phalanx; wrist	PoCUS	Radiography	Sensitivity; specificity; PPV; NPV
Dulchavsky (48)	USA	95	Upper or lower limb	PoCUS	Radiography	Sensitivity; specificity
Hedelin (44)	Sweden	122	Ankle	PoCUS	Radiography	Sensitivity; specificity; PPV; NPV; accuracy; gap in bone
Dallaudière (34)	South America	83	Not specified	PoCUS	Radiography	Sensitivity; specificity
Lau (47)	USA	23	Distal radius	PoCUS	Radiography	Sensitivity; specificity; interrater/intrarater reliability

[3]CT scan used if inconsistency observed between PoCUS and plain radiography
