# Supplementary material for: The effectiveness of ultrasound in the detection of fractures in adults with suspected upper or lower limb injury: a systematic review and subgroup meta-analysis
Source: BMC Emerg Med. 2019 Jan 28;19:17. doi: 10.1186/s12873-019-0226-5 (PMC6350304; doi:10.1186/s12873-019-0226-5)
Supplement: Supplementary file 4 — : Characteristics of Excluded Studies. List of studies excluded at the stage of full-text article review, indicating the reason for study exclusion from the systematic review. (PDF 2357 kb) [file 12873_2019_226_MOESM4_ESM.pdf]

Additional file 4: Characteristics of Excluded Studies: *List of studies excluded at the stage of full-text article review, indicating the reason for study exclusion from the systematic review.*

Study (ref)	Fracture site	Paediatric/adult populations	Non-blinded imaging/operator	Therapeutic ultrasound	Non-clinical study or case series
Avci (49)	Elbow	X			
Baldry (68)	Scaphoid				X
Beltrame (2)	Not specified	X			
Canagasabey (57)	Foot/ankle		X		
Christiansen (62)	Scaphoid			X	
DaCruz (63)	Scaphoid			X	
Dudkiewicz (58)	5th metatarsus		X		
Giladi (64)	Not specified			X	
Hauger (50)	Scaphoid	X			
Jenkins (69)	Scaphoid				X
Kozaci (51)	Metacarpal	X			
Kozaci (52)	Distal radius	X			
McNeil (59)	Not specified		X		
Munk (53)	Scaphoid	X			
Musa (54)	Not specified	X			
Nitz (65)	Medial tibia			X	
Pancione (60)	Hill-Sachs lesion		X		
Papalada (66)	Not specified			X	
Patel (72)	Long bone				X
Senall (55)	Scaphoid	X			
Shenouda (67)	Scaphoid			X	
Sinha (10)	Not specified	X			
Tomer (70)	Not specified				X
Wang (71)	Foot/ankle				X
Waterbrook (56)	Long bone	X			
Yildirim (61)	Scaphoid		X		
		10	5	6	5
