# Supplementary material for: The effectiveness of ultrasound in the detection of fractures in adults with suspected upper or lower limb injury: a systematic review and subgroup meta-analysis
Source: BMC Emerg Med. 2019 Jan 28;19:17. doi: 10.1186/s12873-019-0226-5 (PMC6350304; doi:10.1186/s12873-019-0226-5)

<u>Additional file 5: Summary of Critical Appraisals:</u> Individual summary of critical appraisal following the SIGN methodology checklist [62]

SIGN Checklist (methodology for diagnostic studies)	Papers reviewed																								
	Aksay (2013)	Aksay (2016)	Atilla (2014)	Banal (2009)	Bolandparvaz (2013)	Čičak (1998)	Dallaudière (2015)	Dulchavsky (2002)	Ekinci (2013)	Farin (1996)	Fusetti (2005)	Gungor (2016)	Hedelin (2013)	Herneth (2001)	Javadzadeh (2013)	Kilic (2016)	Kocaoğlu (2016)	Lau (2017)	Marshburn (2004)	Platon (2010)	Sivrikaya (2016)	Tayal (2007)	Tollefson (2016)	Weinberg (2010)	Yesilaras (2013)
1.1 A consecutive sequence or random selection of patients is enrolled.	Y	Y	Y	Y	Y	U	Y	Y	Y	U	Y	Y	Y	Y	Y	Y	Y	Y	Y	Y	Y	Y	Y	Y	Y
1.1 Case-control methods are not used.	Y	Y	Y	N	Y	Y	Y	Y	Y	Y	Y	Y	Y	Y	Y	Y	Y	N	Y	Y	Y	Y	Y	Y	Y
1.2 Inappropriate exclusions are avoided.	Y	Y	Y	Y	Y	U	N	Y	Y	U	Y	Y	U	U	Y	Y	Y	Y	Y	U	Y	Y	N	Y	Y
1.3 The included patients and settings match the key question.	Y	Y	Y	Y	Y	N	Y	Y	Y	N	Y	Y	Y	Y	Y	Y	Y	Y	Y	Y	Y	Y	Y	Y	Y
2.1 Index test interpreted without knowledge of the results of the reference standard.	U	Y	Y	Y	Y	Y	Y	Y	Y	Y	Y	Y	Y	Y	Y	Y	Y	Y	Y	Y	Y	Y	Y	Y	Y
2.2 If a threshold is used, it is pre-specified.	Y	Y	Y	Y	N	Y	N	Y	N	Y	Y	Y	N	Y	Y	Y	N	Y	Y	Y	Y	Y	N	Y	Y
2.3 Index test, its conduct, and its interpretation is similar to that used in the target population.	Y	Y	Y	Y	Y	Y	Y	Y	Y	Y	Y	Y	Y	Y	Y	Y	Y	Y	Y	Y	Y	Y	Y	Y	Y
3.1 The reference standard is likely to correctly identify the target condition.	Y	Y	Y	Y	Y	Y	U	Y	Y	Y	Y	Y	Y	Y	Y	Y	Y	Y	Y	Y	Y	Y	Y	Y	Y
3.2 Reference standard interpreted without knowledge of the results of the index test.	Y	Y	Y	Y	Y	Y	Y	U	Y	Y	Y	Y	Y	U	Y	Y	Y	Y	Y	Y	Y	Y	Y	Y	Y
3.3 Target condition defined by the reference standard matches target population.	Y	Y	Y	Y	Y	Y	Y	Y	Y	Y	Y	Y	Y	Y	Y	Y	Y	Y	Y	Y	Y	Y	Y	Y	Y
4.1 There is an appropriate interval between the index test and the reference standard.	Y	Y	Y	Y	Y	U	U	U	U	U	Y	U	U	Y	U	U	Y	Y	U	Y	U	U	U	U	Y
4.2 All patients receive same reference standard	Y	Y	Y	Y	Y	N	Y	Y	Y	N	Y	Y	Y	Y	Y	Y	Y	Y	N	Y	N	Y	Y	Y	Y
4.3 All patients recruited into the study are included in the analysis	Y	Y	Y	Y	Y	Y	Y	Y	Y	Y	Y	Y	Y	Y	Y		Y	Y	Y	Y	Y	Y	Y	Y	N

Summary of Critical Appraisals: Cochrane Handbook Risk of Bias Summary, which lists individual study bias [32]. Figure constructed using *Review Manager*.

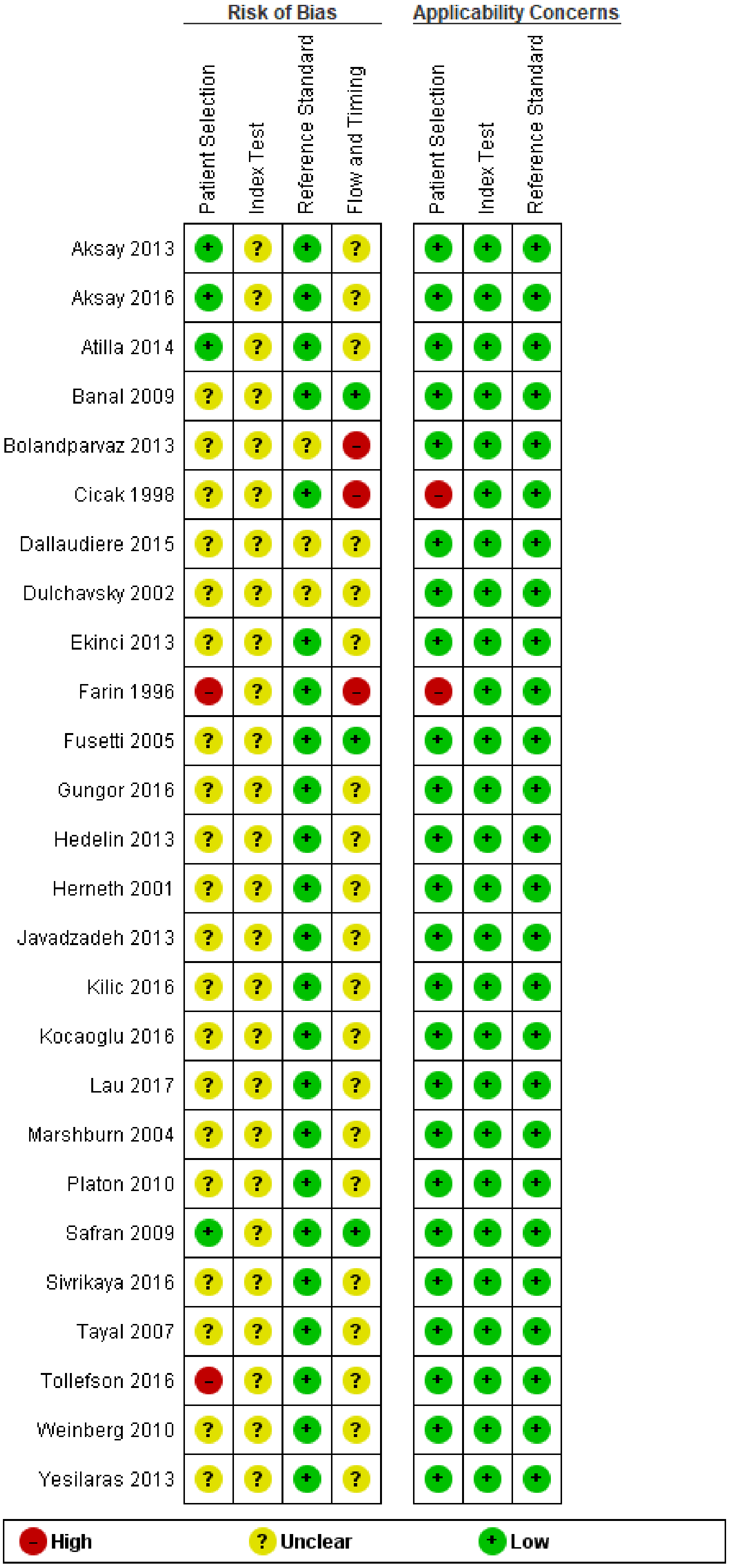

Supplement: Supplementary file 5 — Summary of Critical Appraisals. Individual summary of critical appraisal of included studies following the SIGN methodology checklist. (PDF 3009 kb) [file 12873_2019_226_MOESM5_ESM.pdf]
