# Supplementary material for: The effectiveness of ultrasound in the detection of fractures in adults with suspected upper or lower limb injury: a systematic review and subgroup meta-analysis
Source: BMC Emerg Med. 2019 Jan 28;19:17. doi: 10.1186/s12873-019-0226-5 (PMC6350304; doi:10.1186/s12873-019-0226-5)
Supplement: Supplementary file 6 — Summary of Study Results. Summary tables of primary outcomes extracted from included studies (PDF 3170 kb) [file 12873_2019_226_MOESM6_ESM.pdf]

**Additional file 6: Summary of study results (primary outcomes):**

## Fractures of the hand:

| ref | Study | Fracture site/type | Country | Sensitivity (CI 95%) | Specificity (CI 95%) | NPV (CI 95%) | PPV (CI 95%) |
|---|---|---|---|---|---|---|---|
| (40) | Aksay (2016) | Phalangeal | Turkey | 79.3% (59.7-91.2%) | 90% (81.4-95.0%) | 93.1% (85.0-97.1%) | 71.8% (53.0-85.6%) |
| (39) | Aksay (2013) | 5th metacarpal | Turkey | 97.4% (84.9-99.9%) | 92.9% (79.4-98.1%) | 97.5% (85.3-99.9%) | 92.6% (79.0-98.1%) |
| (43) | Tayal (2007) | Metacarpal; phalangeal | USA | 90% (74-97%) | 98% (95-100%) | 94% (89-99%) | 97% (93-100%) |
| (37) | Gungor (2016) | Distal phalangeal | Turkey | 100% (79-100%) | 98.4% (91-100%) | - | - |
| (36) | Kocaoğlu (2016) | Metacarpal | Turkey | 92.5% (78.5-98%) | 98.28% (89.5-99.9%) | 95% (85-98%) | 97.37% (84.5-99.8%) |
| (42) | Javasdzadeh (2013) | Metacarpal | Iran | 72.73% (43.4-90.3%) | 77.78% (45.3-93.7%) | 70% (39.7-89.2%) | 80% (49.0-94.3%) |
| | | Phalangeal | | 83.33% (60.8-94.2%) | 90.48% (77.9-96.2%) | 92.68% (80.6-97.5%) | 78.95% (56.7-94.5%) |

## Fractures of the scaphoid:

| ref | Study | Fracture site/type | Country | Sensitivity (CI 95%) | Specificity (CI 95%) | NPV (CI 95%) | PPV (CI 95%) |
|---|---|---|---|---|---|---|---|
| (27) | Platon (2010) | Scaphoid | Switzerland | 92% | 71% | 97% | 46% |
| (18) | Fusetti (2005) | Scaphoid | Switzerland | 100% | 79% | 100% | 56% |
| (30) | Herneth (2001) | Scaphoid | Austria | 78% | 100% | 75% | 100% |

## Fractures of the foot and ankle:

| ref | Study | Fracture site/type | Country | Sensitivity (CI 95%) | Specificity (CI 95%) | NPV (CI 95%) | PPV (CI 95%) |
|---|---|---|---|---|---|---|---|
| (45) | Tollefson (2016) | Foot; ankle | USA | 100% (78-100%) | 100% (87-100%) | 100% (89-100%) | 100% (81-100%) |
| (38) | Atilla (2014) | Foot; ankle | Turkey | 87.3% (77.5-93.4%) | 86.4% (93.1-98.2%) | - | - |
| (35) | Yesilaras (2013) | 5th metatarsal | Turkey | 97.1% (82.9-99.8%) | 100% (91.1-100%) | - | - |
| (19) | Ekinci (2013) | Foot; ankle | Turkey | 100% (83.8-100%) | 99.1% (95-99.8%) | 100% (96.4-100%) | 95.2% (89.6-98%) |
| (29) | Banal (2009) | Metatarsal stress | France | 83.3% | 75.9% | 91.7% | 58.8% |
| (44) | Hedelin (2013) | Ankle | Sweden | 100% | 85.9% | 100% | 62.2% |

**Fractures of the upper limb:**

| ref | Study | Fracture site/type | Country | Sensitivity (CI 95%) | Specificity (CI 95%) | NPV (CI 95%) | PPV (CI 95%) |
|---|---|---|---|---|---|---|---|
| (41) | Bolandparvaz (2013) | Upper limb | Iran | 55.5% (22.6-84.6%) | 84% (53.6-97.2%) | 73% (44.8-91.0%) | 71% (30.2-94.8%) |
| (32) | Marshburn (2004) | Humeral; femoral | USA | 93% (77-99%) | 83% (74-98%) | - | - |
| (33) | Sivrikaya (2016) | Radial | Turkey | 100% (92.4-100%) | 88.2% (71.6-96.1%) | - | - |
| | | Ulnar | | 89.5% (65.5-98.1%) | 94.6% (86-98.2%) | - | - |
| (42) | Javadzadeh (2014) | Wrist | Iran | 42.11% (23.1-63.7%) | 86.96% (74.3-98.9%) | 78.43% (65.4-87.5%) | 57.14% (32.6-78.6%) |
| | | Distal forearm | | 89.06% (79.1-94.6%) | 94.41% (81.5-95.3%) | 90.41% (81.5-95.3%) | 93.44% (84.3-97.4%) |
| (48) | Dulchavsky (2002) | Forearm; arm | USA | 92% | 100% | - | - |
| (34) | Dallaudière (2015) | Upper limb | South America | 96.9% | 100% | 93.3% | 100% |
| (47) | Lau (2016) | Distal radial | USA | 100% | 90-95% | - | - |

**Fractures of the lower limb:**

| ref | Study | Fracture site/type | Country | Sensitivity (CI 95%) | Specificity (CI 95%) | NPV (CI 95%) | PPV (CI 95%) |
|---|---|---|---|---|---|---|---|
| (41) | Bolandparvaz (2013) | Lower limb | Iran | 75% (35.5-95.5%) | 72% (39.3-92.6%) | 80% (44.2-96.4%) | 66% (30.9-90%) |
| (31) | Safran (2009) | Hip | Israel | 100% | 65% | 100% | 59% |
| (32) | Marshburn (2004) | Humeral; femoral | USA | 93% (77-99%) | 83% (74-98%) | - | - |
| (26) | Kilic (2016) | Patella | Turkey | 93.3% (66-99.7%) | 94.8% (86.5-98.3%) | 98.5% (91.7-99.9%) | 77.8% (51.9-92.6%) |
| (48) | Dulchavsky (2002) | Femoral; tibia/fibula | USA | 83% | 100% | - | - |
| (34) | Dallaudière (2015) | Lower limb | South America | 100% | 80% | 100% | 93.8% |

**Other fracture sites:**

| ref | Study | Fracture site/type | Country | Sensitivity (CI 95%) | Specificity (CI 95%) |
|---|---|---|---|---|---|
| (28) | Weinberg (2010) | Not specified | USA | 60% (39-78%) | 92% (87-96%) |
| (46) | Čičak (1998) | Hill-Sachs lesion (shoulder) | Croatia | 96% | 100% |
| (25) | Farin (1996) | Hill-Sachs lesion (shoulder) | Finland | 91% | 95% |
| (48) | Dulchavsky (2002) | Hand/foot | USA | 50% | 100% |
