# Supplementary material for: The effectiveness of ultrasound in the detection of fractures in adults with suspected upper or lower limb injury: a systematic review and subgroup meta-analysis
Source: BMC Emerg Med. 2019 Jan 28;19:17. doi: 10.1186/s12873-019-0226-5 (PMC6350304; doi:10.1186/s12873-019-0226-5)

Additional file 7: Meta-analysis of pooled upper limb fracture data:

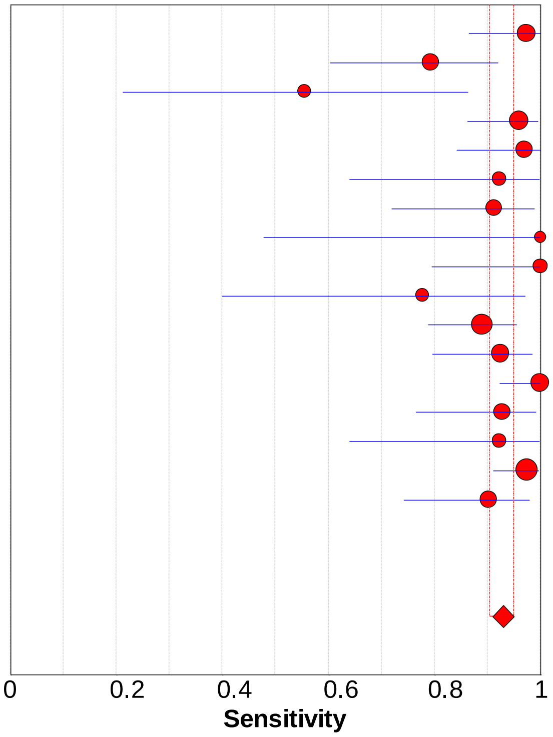

	Sensitivity (95% CI)	
Aksay 2013	0.97	(0.87 - 1.00)
Aksay 2016	0.79	(0.60 - 0.92)
Bolandparvaz 2013	0.56	(0.21 - 0.86)
Cicak 1998	0.96	(0.86 - 1.00)
Dallaudière 2015	0.97	(0.84 - 1.00)
Dulchavsky 2002	0.92	(0.64 - 1.00)
Farin 1996	0.91	(0.72 - 0.99)
Fusetti 2005	1.00	(0.48 - 1.00)
Gungor 2016	1.00	(0.79 - 1.00)
Herneth 2001	0.78	(0.40 - 0.97)
Javadzadeh 2013	0.89	(0.79 - 0.95)
Kocaoglu 2016	0.93	(0.80 - 0.98)
Lau 2017	1.00	(0.92 - 1.00)
Marshburn 2004	0.93	(0.76 - 0.99)
Platon 2010	0.92	(0.64 - 1.00)
Sivrikaya 2016	0.97	(0.91 - 1.00)
Tayal 2007	0.90	(0.74 - 0.98)

Pooled Sensitivity = 0.93 (0.90 to 0.95)
Chi-square = 35.32; df = 16 (p = 0.0036)
Inconsistency (I-square) = 54.7 %

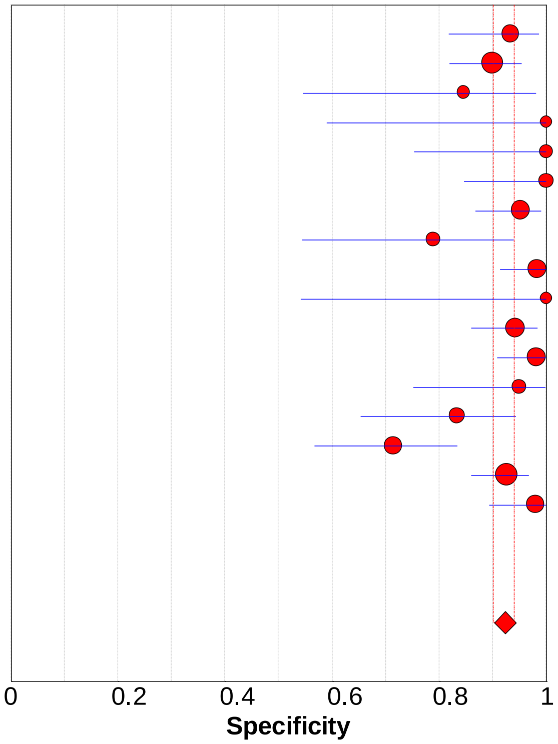

	Specificity (95% CI)	
Aksay 2013	0.93	(0.82 - 0.99)
Aksay 2016	0.90	(0.82 - 0.95)
Bolandparvaz 2013	0.85	(0.55 - 0.98)
Cicak 1998	1.00	(0.59 - 1.00)
Dallaudière 2015	1.00	(0.75 - 1.00)
Dulchavsky 2002	1.00	(0.85 - 1.00)
Farin 1996	0.95	(0.87 - 0.99)
Fusetti 2005	0.79	(0.54 - 0.94)
Gungor 2016	0.98	(0.91 - 1.00)
Herneth 2001	1.00	(0.54 - 1.00)
Javadzadeh 2013	0.94	(0.86 - 0.98)
Kocaoglu 2016	0.98	(0.91 - 1.00)
Lau 2017	0.95	(0.75 - 1.00)
Marshburn 2004	0.83	(0.65 - 0.94)
Platon 2010	0.71	(0.57 - 0.83)
Sivrikaya 2016	0.93	(0.86 - 0.97)
Tayal 2007	0.98	(0.89 - 1.00)

Pooled Specificity = 0.92 (0.90 to 0.94)
Chi-square = 47.52; df = 16 (p = 0.0001)
Inconsistency (I-square) = 66.3 %

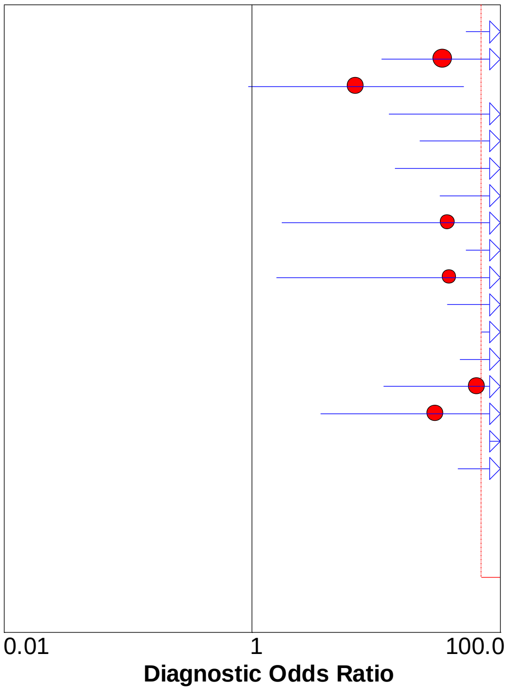

	Diagnostic OR (95% CI)
Aksay 2013	532.00 (53.05 - 5,334.61)
Aksay 2016	34.50 (11.12 - 107.02)
Bolandparvaz 2013	6.88 (0.93 - 50.78)
Cicak 1998	291.00 (12.69 - 6,671.29)
Dallaudière 2015	585.00 (22.39 - 15,283.68)
Dulchavsky 2002	375.00 (14.19 - 9,910.73)
Farin 1996	210.00 (32.79 - 1,344.85)
Fusetti 2005	37.89 (1.74 - 823.94)
Gungor 2016	1,353.00 (52.65 - 34,769.58
Herneth 2001	39.00 (1.57 - 969.19)
Javadzadeh 2013	134.36 (37.41 - 482.55)
Kocaoglu 2016	703.00 (70.43 - 7,016.72)
Lau 2017	1,209.00 (47.16 - 30,994.14
Marshburn 2004	65.00 (11.53 - 366.38)
Platon 2010	30.00 (3.56 - 252.97)
Sivrikaya 2016	475.00 (98.04 - 2,301.35)
Tayal 2007	457.33 (45.38 - 4,608.84)

Random Effects Model
Pooled Diagnostic Odds Ratio = 145.46 (70.73 to 299.17)
Cochran-Q = 29.85; df = 16 (p = 0.0188)
Inconsistency (I-square) = 46.4 %
Tau-squared = 0.9639

Study	TP	FP	FN	TN	Sensitivity (95% CI)	Specificity (95% CI)
Aksay 2013	38	3	1	42	0.97 [0.87, 1.00]	0.93 [0.82, 0.99]
Aksay 2016	23	9	6	81	0.79 [0.60, 0.92]	0.90 [0.82, 0.95]
Bolandparvaz 2013	5	2	4	11	0.56 [0.21, 0.86]	0.85 [0.55, 0.98]
Cicak 1998	48	0	2	7	0.96 [0.86, 1.00]	1.00 [0.59, 1.00]
Dallaudiere 2015	32	0	1	13	0.97 [0.84, 1.00]	1.00 [0.75, 1.00]
Dulchavsky 2002	12	0	1	22	0.92 [0.64, 1.00]	1.00 [0.85, 1.00]
Farin 1996	21	3	2	60	0.91 [0.72, 0.99]	0.95 [0.87, 0.99]
Fusetti 2005	5	4	0	15	1.00 [0.48, 1.00]	0.79 [0.54, 0.94]
Gungor 2016	16	1	0	61	1.00 [0.79, 1.00]	0.98 [0.91, 1.00]
Herneth 2001	7	0	2	6	0.78 [0.40, 0.97]	1.00 [0.54, 1.00]
Javadzadeh 2013	57	4	7	66	0.89 [0.79, 0.95]	0.94 [0.86, 0.98]
Kocaoglu 2016	37	1	3	57	0.93 [0.80, 0.98]	0.98 [0.91, 1.00]
Lau 2017	46	1	0	19	1.00 [0.92, 1.00]	0.95 [0.75, 1.00]
Marshburn 2004	26	5	2	25	0.93 [0.76, 0.99]	0.83 [0.65, 0.94]
Platon 2010	12	14	1	35	0.92 [0.64, 1.00]	0.71 [0.57, 0.83]
Sivrikaya 2016	76	8	2	100	0.97 [0.91, 1.00]	0.93 [0.86, 0.97]
Tayal 2007	28	1	3	49	0.90 [0.74, 0.98]	0.98 [0.89, 1.00]

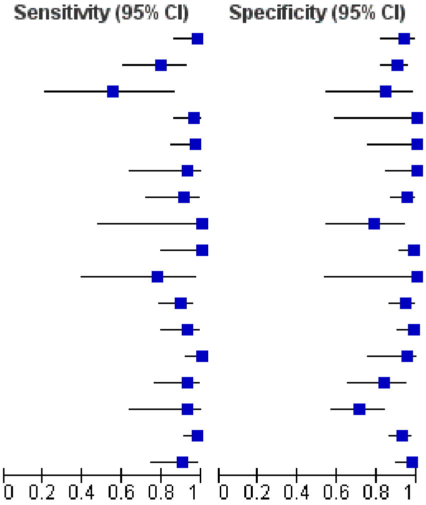

Supplement: Supplementary file 7 — Meta-analysis of pooled upper limb fracture data. Meta-analysis tables produced from the pooled subgroup data relating to upper limb fracture detection. (PDF 268 kb) [file 12873_2019_226_MOESM7_ESM.pdf]
