# Supplementary material for: The effectiveness of ultrasound in the detection of fractures in adults with suspected upper or lower limb injury: a systematic review and subgroup meta-analysis
Source: BMC Emerg Med. 2019 Jan 28;19:17. doi: 10.1186/s12873-019-0226-5 (PMC6350304; doi:10.1186/s12873-019-0226-5)

**Additional file 8: Meta-analysis of pooled lower limb fracture data:**

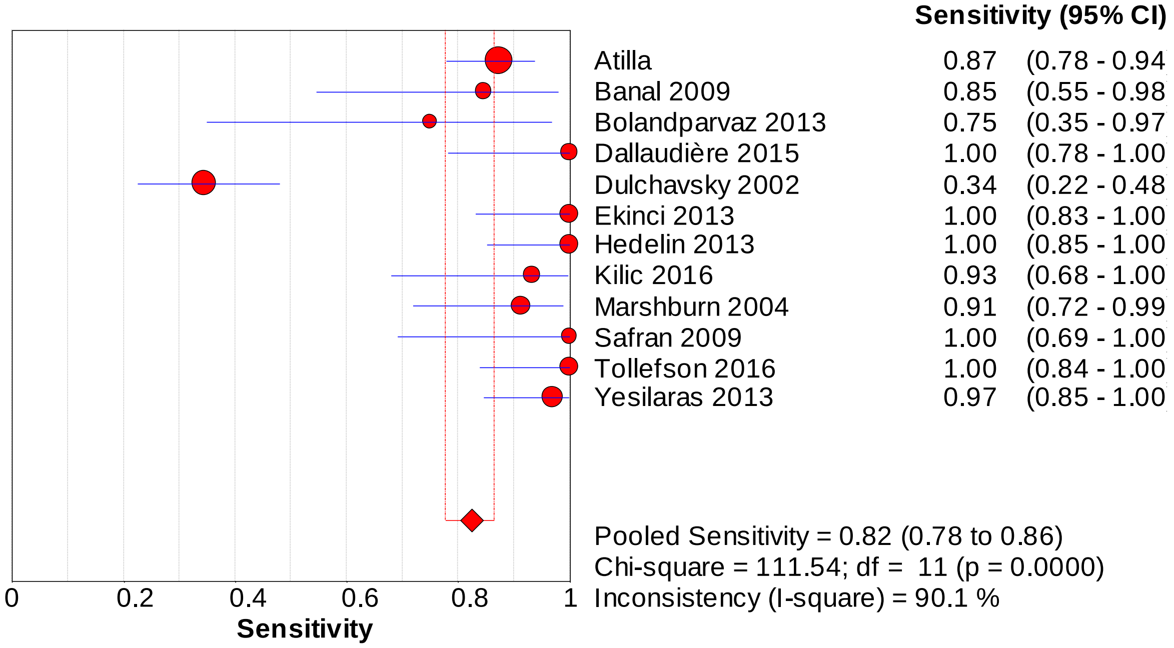


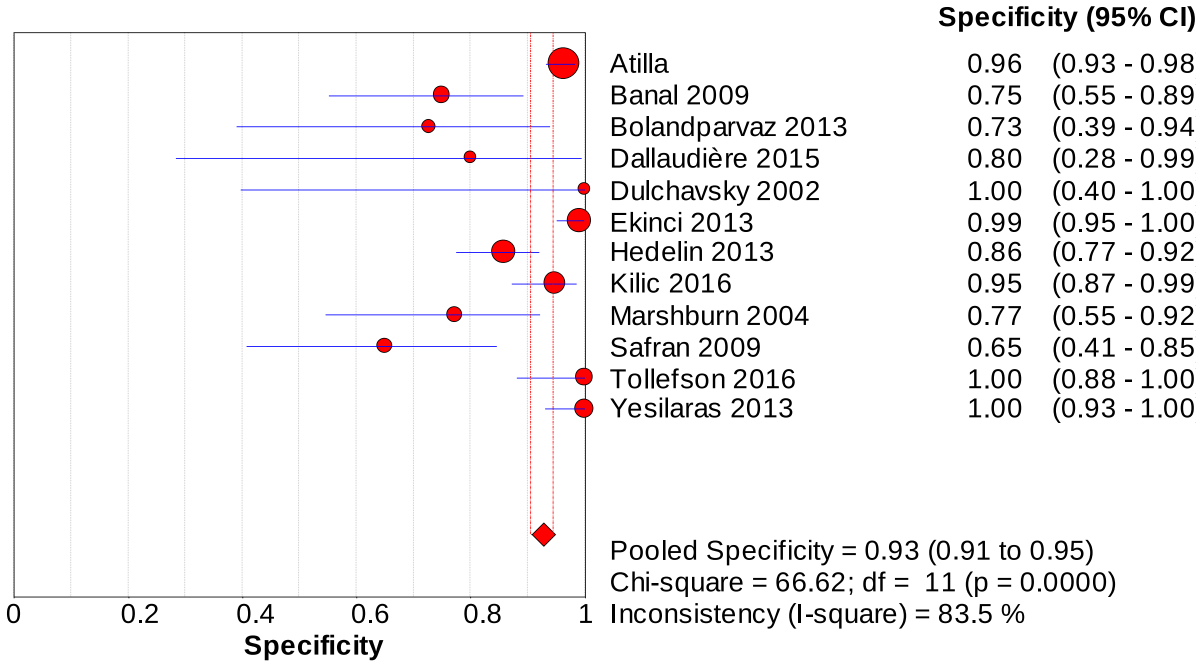

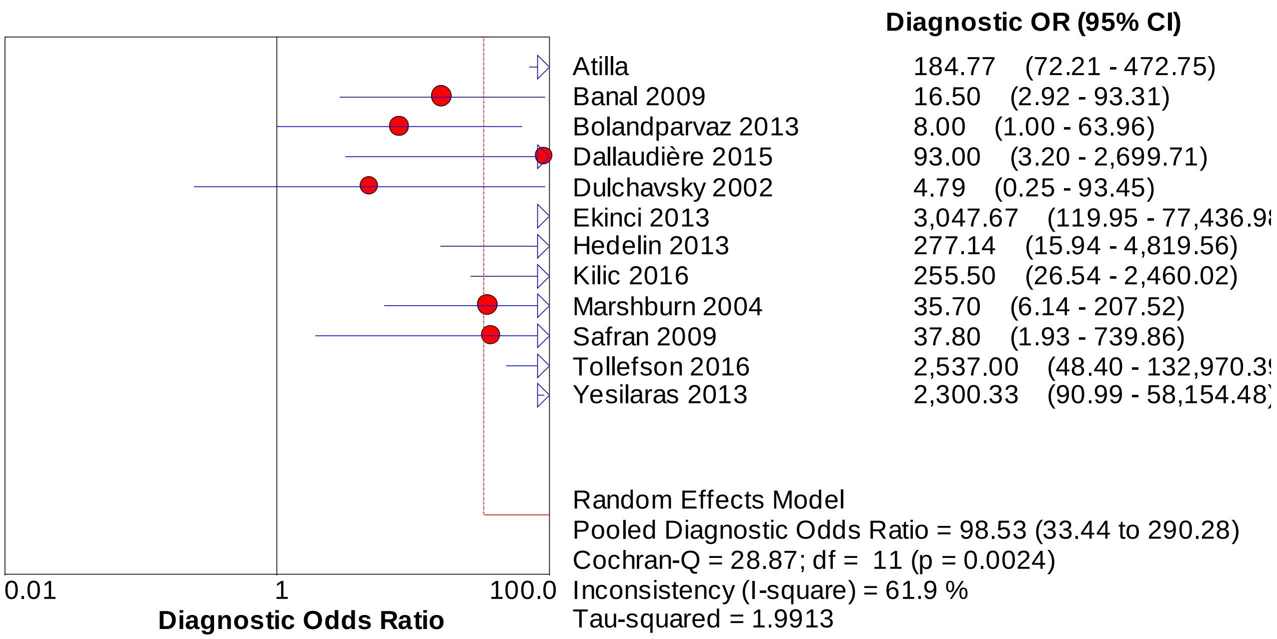


| Study | TP | FP | FN | TN | Sensitivity (95% CI) | Specificity (95% CI) |
|---|---|---|---|---|---|---|
| Atilla 2014 | 69 | 9 | 10 | 241 | 0.87 [0.78, 0.94] | 0.96 [0.93, 0.98] |
| Banal 2009 | 11 | 7 | 2 | 21 | 0.85 [0.55, 0.98] | 0.75 [0.55, 0.89] |
| Bolandparvaz 2013 | 6 | 3 | 2 | 8 | 0.75 [0.35, 0.97] | 0.73 [0.39, 0.94] |
| Dallaudiere 2015 | 15 | 1 | 0 | 4 | 1.00 [0.78, 1.00] | 0.80 [0.28, 0.99] |
| Dulchavsky 2002 | 20 | 0 | 38 | 4 | 0.34 [0.22, 0.48] | 1.00 [0.40, 1.00] |
| Ekinci 2013 | 20 | 1 | 0 | 111 | 1.00 [0.83, 1.00] | 0.99 [0.95, 1.00] |
| Hedelin 2013 | 23 | 14 | 0 | 85 | 1.00 [0.85, 1.00] | 0.86 [0.77, 0.92] |
| Kilic 2016 | 14 | 4 | 1 | 73 | 0.93 [0.68, 1.00] | 0.95 [0.87, 0.99] |
| Marshburn 2004 | 21 | 5 | 2 | 17 | 0.91 [0.72, 0.99] | 0.77 [0.55, 0.92] |
| Safran 2009 | 10 | 7 | 0 | 13 | 1.00 [0.69, 1.00] | 0.65 [0.41, 0.85] |
| Tollefson 2016 | 21 | 0 | 0 | 29 | 1.00 [0.84, 1.00] | 1.00 [0.88, 1.00] |
| Yesilaras 2013 | 33 | 0 | 1 | 51 | 0.97 [0.85, 1.00] | 1.00 [0.93, 1.00] |

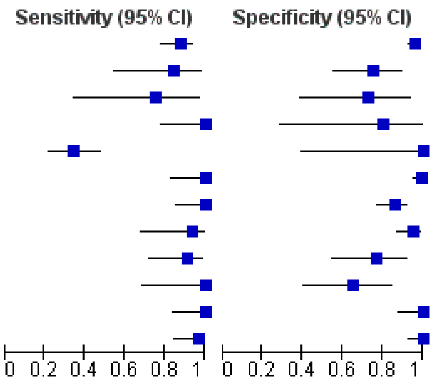

Supplement: Supplementary file 8 — Meta-analysis of pooled lower limb fracture data. Meta-analysis tables produced from the pooled subgroup data relating to lower limb fracture detection. (PDF 269 kb) [file 12873_2019_226_MOESM8_ESM.pdf]
